# Taxonomy of Linyphiidae (Arthropoda, Araneae) in Théniet El Had National Park with new records for Algeria

**DOI:** 10.3897/BDJ.12.e125331

**Published:** 2024-09-18

**Authors:** Amina Saadi, Badis Bakhouche, Imad Djemadi, Lynda Beladjal, Ourida Kherbouche-Abrous

**Affiliations:** 1 Dynamic and Biodiversity Laboratory, Faculty of Biological Sciences, University of Sciences and Technology Houari Boumediene, BP 32 El Alia, Bab Ezzouar, 16024, Algiers, Algeria Dynamic and Biodiversity Laboratory, Faculty of Biological Sciences, University of Sciences and Technology Houari Boumediene, BP 32 El Alia, Bab Ezzouar, 16024 Algiers Algeria; 2 Laboratory of biological and oceanography and the marine environment, Faculty of Biological Sciences, University of Sciences and Technology Houari Boumediene, BP 32 El Alia, Bab Ezzouar, 16024, Algiers, Algeria Laboratory of biological and oceanography and the marine environment, Faculty of Biological Sciences, University of Sciences and Technology Houari Boumediene, BP 32 El Alia, Bab Ezzouar, 16024 Algiers Algeria; 3 Environment research center, environment and biodiversity division, Annaba, Algeria Environment research center, environment and biodiversity division Annaba Algeria; 4 Ghent University, Department of Biology, Terrestrial Ecology Unit, K.L. Ledeganckstraat 35, B-9000, Ghent, Belgium Ghent University, Department of Biology, Terrestrial Ecology Unit, K.L. Ledeganckstraat 35, B-9000 Ghent Belgium

## Abstract

**Background:**

*Cedrusatlantica*, a species of coniferous tree in the Pinaceae family, is endemic to Algeria and Morocco. It originated in the Atlas Mountains of North Africa, hence the name Atlas Cedar. Cedar forests are home to a diverse range of flora and fauna. Yet despite their importance as a rich reservoir of fauna, very few faunistic studies have been carried out in Algeria in this type of ecosystem, particularly for the Araneae. Théniet El Had National Park (34.25 km^2^) is a unique national park in the western part of Algeria with centuries-old cedars. The present work is the first of its kind, consisting of establishing a preliminary list of Lyniphiidae in this typical ecosystem.

**New information:**

A preliminary checklist of Linyphiidae of the National Park is provided, following a sampling period between January and December 2019. A total of 24 species were collected belonging to 15 genera of which *Pelecopsis* is the richest genus with six species. *Pelecopsisoujda* Bosmans & Abrous, 1992 and *Walckenaeriaheimbergi* Bosmans, 2007, only known from Morocco so far, were collected for the first time in Algeria. Their distribution area is, thus, more extensive than present.

## Introduction

Linyphiidae is the second richest family of spiders, with 4847 species belonging to 634 genera (World Spider Catalog 2024). Although new species are still being described all over the world, this family is still less known. This can be explained not only by the small size of most individuals (1 to 8.5 mm), hence the name dwarf spiders, but their complex genitalia, and species richness are the other reasons for their difficult identification.

**Figure 1.**
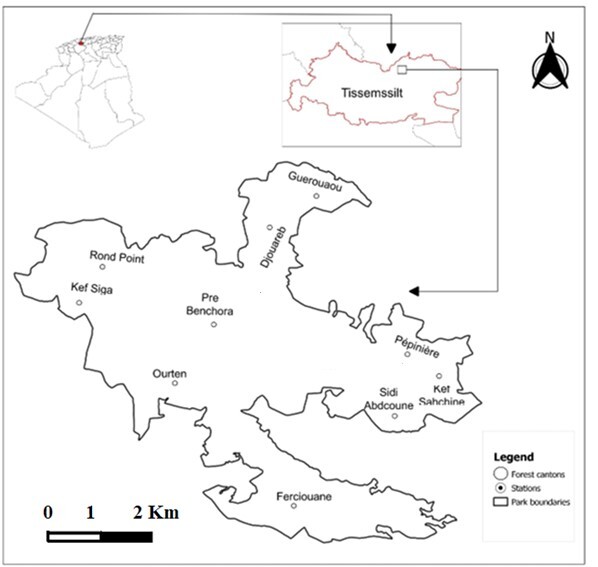
Location of Théniet El Had National Park.

In Algeria, 57 genera and 143 species are identified and described in the following papersBosmans 1985a, Bosmans 1985b, Bosmans 1986, Bosmans and Abrous 1990, Bosmans 1991a, Bosmans 1991b, Bosmans and Abrous 1992, Bosmans and Bouragba 1992, Bosmans and Chergui 1993, Bosmans and DeSmet 1993, Bosmans 1996, Bosmans 2002, Bosmans 2006a, Bosmans 2006b, Bosmans 2007, Bosmans 2008, World Spider Catalog 2024. Despite this species richness both in Algeria and elsewhere, the biogeography and the ecological preferences of this family are still less known. The present study aims not only to identify the Lyniphiidae in Theniet El Had National Park (THNP) (Fig. 1), but also to study the distribution of species in this typical ecosystem. In addition, we report here new records of *Pelecopsisoujda* Bosmans & Abrous, 1992 and *Walckenaeriaheimbergi* Bosmans, 2007 from THNP and consequently in Algeria, so far known from Morocco.

## Materials and methods

### Study area

Théniet El Had National Park (THNP) is one of the 11 national parks of Algeria, located in the Wilaya of Tissemsilt. It has several forests and it is located at the foothills of the Ouarsenis Mountain chain in the Tell Atlas and covering an area of 3425 ha (north-west Algeria, 35°47'-35°54'N, 01°54'-02°02'E) (Fig. 1). The Park created by the Algerian government in 1983, has a northwest-southeast orientated valley with extremely steep slopes that can exceed 50% in the northern part (Sarmoum et al. 2018). The altitude is between 854 and 1787 m a.s.l. with a forest coverage of 86.7%. The cedar forest in this Park is unique in western Algeria. It is also one of the rare places in the Mediterranean region where the cork oak grows at more than 1600 m. The flora is very varied, with many endemic species to Algeria.

### Sampling methods

Linyphiidae were collected using two sampling methods: pitfalls traps and hand collecting. The pitfalls were PET plastic bottles (diameter 8 cm, height 18 cm). The top of the bottle was cut off and used as a funnel for the trap. Ten traps were hollowed out in a straight line at 1 m intervals at each exposure level. The top of each trap was even with the ground surface. A solution of formaldehyde (4%) with a little detergent to decrease surface pressure was used as a fixative. The traps were emptied once a month for a full year. Hand-collecting method is a visual search where spiders are hand-collected by overturning objects on the ground such as stones, branches etc. or by picking up spiders from trees, bushes etc.

After sorting, spiders were preserved in 70% ethanol and identified in the laboratory using a stereomicroscope (Optika SZM-1), based on the following works:Millidge 1981, Bosmans 1985a, Bosmans 1985b, Bosmans and Abrous 1990, Bosmans 1991a, Bosmans 1991b, Bosmans and Abrous 1992, Bosmans and Chergui 1993, Bosmans and DeSmet 1993, Wunderlich 1994, Bosmans 2006a, Bosmans 2006b, Bosmans 2007, Bosmans 2008, Dawson et al. 2011 following the nomenclature of the World Spider Catalog (2024). The material collected was deposited in the laboratory of Dynamics and Biodiversity, University of Sciences and Technology Houari Boumediene, Algiers, Algeria.

## Taxon treatments

### Agyneta pseudorurestris

Wunderlich, 1980

7A5C3138-9DF1-54C1-848F-6F41600F1AF0

#### Materials

- **Type status:** Other material. **Occurrence:** recordedBy: Amina Saadi; individualCount: 1; sex: female; lifeStage: adult; occurrenceID: 907DFAAB-94F8-5DB8-9E97-FC67DE044D38; **Taxon:** scientificNameID: urn:lsid:nmbe.ch:spidersp:011703; scientificName: *Agynetapseudorurestris* Wunderlich, 1980; kingdom: Animalia; phylum: Arthropoda; class: Arachnida; order: Araneae; family: Linyphiidae; genus: Agyneta; taxonRank: species; taxonomicStatus: accepted; **Location:** higherGeography: North Africa; continent: Africa; country: Algeria; countryCode: DZ; municipality: Theniet El Had; verbatimElevation: 1448 m; verbatimCoordinateSystem: 35°51’30’’ N, 01°59’11’’ E; **Identification:** identifiedBy: Ourida Kherbouche-Abrous; **Event:** samplingProtocol: pitfall traps; year: 2019; month: 2; day: 1-15
- **Type status:** Other material. **Occurrence:** recordedBy: Amina Saadi; individualCount: 1; sex: male; lifeStage: adult; occurrenceID: 8C235709-ACAE-57C8-862D-D4EE381DD4AB; **Taxon:** scientificNameID: urn:lsid:nmbe.ch:spidersp:011703; scientificName: *Agynetapseudorurestris* Wunderlich, 1980; kingdom: Animalia; phylum: Arthropoda; class: Arachnida; order: Araneae; family: Linyphiidae; genus: Agyneta; taxonRank: species; taxonomicStatus: accepted; **Location:** higherGeography: North Africa; continent: Africa; country: Algeria; countryCode: DZ; municipality: Theniet El Had; verbatimElevation: 1470 m; verbatimCoordinateSystem: 35°52’00’’ N, 01°58’07’’ E; **Identification:** identifiedBy: Ourida Kherbouche-Abrous; **Event:** samplingProtocol: pitfall traps; year: 2019; month: 5; day: 1-15
- **Type status:** Other material. **Occurrence:** recordedBy: Badis Bakhouche; individualCount: 1; sex: male; lifeStage: adult; occurrenceID: 17F90CE1-4196-50F1-9196-0C13909C8DBA; **Taxon:** scientificNameID: urn:lsid:nmbe.ch:spidersp:011703; scientificName: *Agynetapseudorurestris* Wunderlich, 1980; kingdom: Animalia; phylum: Arthropoda; class: Arachnida; order: Araneae; family: Linyphiidae; genus: Agyneta; taxonRank: species; taxonomicStatus: accepted; **Location:** higherGeography: North Africa; country: Algeria; countryCode: DZ; municipality: Theniet El Had; verbatimElevation: 1470 m; verbatimCoordinateSystem: 35°52’00’’ N, 01°58’07’’ E; **Identification:** identifiedBy: Ourida Kherbouche-Abrous; **Event:** samplingProtocol: hand collecting; year: 2019; month: 7; day: 1-15

#### Distribution

**Algerian distribution**: Almost the whole of the northern part of the country, from the east to the west (Bosmans 2006b).

**Global distribution**: Mediterranean (World Spider Catalog 2024).

### Alioranus pauper

(Simon, 1882)

938D9B7E-15BA-55DC-9E05-743671154063

#### Materials

- **Type status:** Other material. **Occurrence:** recordedBy: Badis Bakhouche; individualCount: 1; sex: male; lifeStage: adult; occurrenceID: 6072F4EE-EA65-5C1E-8AE6-A941F35694B2; **Taxon:** scientificNameID: urn:lsid:nmbe.ch:spidersp:009378; scientificName: *Alioranuspauper* (Simon, 1882); kingdom: Animalia; phylum: Arthropoda; class: Arachnida; order: Araneae; family: Linyphiidae; genus: Alioranus; taxonRank: species; taxonomicStatus: accepted; **Location:** higherGeography: North Africa; continent: Africa; country: Algeria; countryCode: DZ; municipality: Theniet El Had; verbatimElevation: 1474 m; verbatimCoordinateSystem: 35°51’58’’ N, 01°58’06’’E; **Identification:** identifiedBy: Ourida Kherbouche-Abrous; **Event:** samplingProtocol: hand collecting; year: 2019; month: 3; day: 1-15

#### Distribution

**Algerian distribution**: From the coastal region to the interior of the country, especially the east and south-east, found also in the north-eastern Sahara (Bosmans 2007).

**Global distribution**: Mediterranean (World Spider Catalog 2024).

### Centromerus cinctus

(Simon, 1884)

3C14705C-D726-5739-B995-81B6BBCBDFC6

#### Materials

- **Type status:** Other material. **Occurrence:** recordedBy: Amina Saadi; individualCount: 1; sex: male; lifeStage: adult; occurrenceID: 0A24B19E-75B6-51B7-B507-B72D4F4B7964; **Taxon:** scientificNameID: urn:lsid:nmbe.ch:spidersp:009723; scientificName: *Centromeruscinctus* (Simon, 1884); kingdom: Animalia; phylum: Arthropoda; class: Arachnida; order: Araneae; family: Linyphiidae; genus: Centromerus; taxonRank: species; taxonomicStatus: accepted; **Location:** higherGeography: North Africa; continent: Africa; country: Algeria; countryCode: DZ; municipality: Theniet El Had; verbatimElevation: 1448 m; verbatimCoordinateSystem: 35°51’30’’ N, 01°59’11’’ E; **Identification:** identifiedBy: Ourida Kherbouche-Abrous; **Event:** samplingProtocol: pitfall traps; year: 2019; month: 4; day: 1-15
- **Type status:** Other material. **Occurrence:** recordedBy: Amina Saadi; individualCount: 1; sex: female; lifeStage: adult; occurrenceID: A1C32671-9346-50FA-8C5F-E84E4F56CF30; **Taxon:** scientificNameID: urn:lsid:nmbe.ch:spidersp:009723; scientificName: *Centromeruscinctus* (Simon, 1884); kingdom: Animalia; phylum: Arthropoda; class: Arachnida; order: Araneae; family: Linyphiidae; genus: Centromerus; taxonRank: species; taxonomicStatus: accepted; **Location:** higherGeography: North Africa; continent: Africa; country: Algeria; countryCode: DZ; municipality: Theniet El Had; verbatimElevation: 1470 m; verbatimCoordinateSystem: 35°52’00’’ N, 01°58’07’’ E; **Identification:** identifiedBy: Ourida Kherbouche-Abrous; **Event:** samplingProtocol: pitfall traps; year: 2019; month: 12; day: 16-31

#### Distribution

**Algerian distribution**: North, north-east and south-west of the country (Bosmans 1986, Bosmans 2006b).

**Global distribution**: France (Corsica), Algeria, Tunisia. (World Spider Catalog 2024).

### Centromerus sinuatus

Bosmans, 1986

849BB2C4-2A0D-577D-8E77-F697A2BF022C

#### Materials

- **Type status:** Other material. **Occurrence:** recordedBy: Amina Saadi; individualCount: 2; sex: male; lifeStage: adult; occurrenceID: C27C68C9-3C41-5182-A445-90C62F17BBD6; **Taxon:** scientificNameID: urn:lsid:nmbe.ch:spidersp:009773; scientificName: *Centromerussinuatus* Bosmans, 1986; kingdom: Animalia; phylum: Arthropoda; class: Arachnida; order: Araneae; family: Linyphiidae; genus: Centromerus; taxonRank: species; taxonomicStatus: accepted; **Location:** higherGeography: North Africa; continent: Africa; country: Algeria; countryCode: DZ; municipality: Theniet El Had; verbatimElevation: 1449 m; verbatimCoordinateSystem: 35°51’34’’ N, 01°58’50’’ E; **Identification:** identifiedBy: Ourida Kherbouche-Abrous; **Event:** samplingProtocol: pitfall traps; year: 2019; month: 1; day: 1-15
- **Type status:** Other material. **Occurrence:** recordedBy: Badis Bakhouche; Imad Djemadi; individualCount: 13; sex: 8 males, 6 females; lifeStage: adult; occurrenceID: 181D0509-FF1B-591B-9C8E-B5561897D349; **Taxon:** scientificNameID: urn:lsid:nmbe.ch:spidersp:009773; scientificName: *Centromerussinuatus* Bosmans, 1986; kingdom: Animalia; phylum: Arthropoda; class: Arachnida; order: Araneae; family: Linyphiidae; genus: Centromerus; taxonRank: species; taxonomicStatus: accepted; **Location:** higherGeography: North Africa; continent: Africa; country: Algeria; countryCode: DZ; municipality: Theniet El Had; verbatimElevation: 1456 m; verbatimCoordinateSystem: 35°51’18’’ N, 01°59’20’’ E; **Identification:** identifiedBy: Ourida Kherbouche-Abrous; **Event:** samplingProtocol: hand collecting; year: 2019; month: 4; day: 1-15
- **Type status:** Other material. **Occurrence:** recordedBy: Amina Saadi; individualCount: 2; sex: male, 1 female; lifeStage: adult; occurrenceID: 23C067FB-5A7B-526F-A5FB-F899D2A6AA02; **Taxon:** scientificNameID: urn:lsid:nmbe.ch:spidersp:009773; scientificName: *Centromerussinuatus* Bosmans, 1986; kingdom: Animalia; phylum: Arthropoda; class: Arachnida; order: Araneae; family: Linyphiidae; genus: Centromerus; taxonRank: species; taxonomicStatus: accepted; **Location:** higherGeography: North Africa; continent: Africa; country: Algeria; countryCode: DZ; municipality: Theniet El Had; verbatimElevation: 1449 m; verbatimCoordinateSystem: 35°51’34’’ N, 01°58’50’’ E; **Identification:** identifiedBy: Ourida Kherbouche-Abrous; **Event:** samplingProtocol: pitfall traps; year: 2019; month: 12; day: 16-31

#### Distribution

**Algerian distribution**: North part of the country (Bosmans 1986, Bosmans 2006a).

**Global distribution**: Morocco, Algeria, Tunisia (World Spider Catalog 2024).

### Centromerus succinus

(Simon, 1884)

472154A2-E4CA-533A-AB25-2856BDEF7407

#### Materials

- **Type status:** Other material. **Occurrence:** recordedBy: Amina Saadi; individualCount: 2; sex: 2 males; lifeStage: adult; occurrenceID: B7B77EEC-5533-5A58-BE59-65D1F6E92594; **Taxon:** scientificNameID: urn:lsid:nmbe.ch:spidersp:009777; scientificName: *Centromerussuccinus* (Simon, 1884); kingdom: Animalia; phylum: Arthropoda; class: Arachnida; order: Araneae; family: Linyphiidae; genus: Centromerus; taxonRank: species; taxonomicStatus: accepted; **Location:** higherGeography: North Africa; continent: Africa; country: Algeria; countryCode: DZ; municipality: Theniet El Had; verbatimElevation: 1456 m; verbatimCoordinateSystem: 35°51’18’’ N, 01°59’20’’ E; **Identification:** identifiedBy: Ourida Kherbouche-Abrous; **Event:** samplingProtocol: pitfall traps; year: 2019; month: 1; day: 1-15
- **Type status:** Other material. **Occurrence:** recordedBy: Amina Saadi; individualCount: 4; sex: 1 male, 3 females; lifeStage: adult; occurrenceID: 82CA302E-8061-5D91-A781-35FA1DCA11A8; **Taxon:** scientificNameID: urn:lsid:nmbe.ch:spidersp:009777; scientificName: *Centromerussuccinus* (Simon, 1884); kingdom: Animalia; phylum: Arthropoda; class: Arachnida; order: Araneae; family: Linyphiidae; genus: Centromerus; taxonRank: species; taxonomicStatus: accepted; **Location:** higherGeography: North Africa; continent: Africa; country: Algeria; countryCode: DZ; municipality: Theniet El Had; verbatimElevation: 1448 m; verbatimCoordinateSystem: 35°51’30’’ N, 01°59’11’’ E; **Identification:** identifiedBy: Ourida Kherbouche-Abrous; **Event:** samplingProtocol: pitfall traps; year: 2019; month: 2; day: 1-15
- **Type status:** Other material. **Occurrence:** recordedBy: Amina Saadi; individualCount: 1; sex: male; lifeStage: adult; occurrenceID: B48347A1-E25A-5D95-9E0A-75C4D91F8AF1; **Taxon:** scientificNameID: urn:lsid:nmbe.ch:spidersp:009777; scientificName: *Centromerussuccinus* (Simon, 1884); kingdom: Animalia; phylum: Arthropoda; class: Arachnida; order: Araneae; family: Linyphiidae; genus: Centromerus; taxonRank: species; taxonomicStatus: accepted; **Location:** higherGeography: North Africa; continent: Africa; country: Algeria; countryCode: DZ; municipality: Theniet El Had; verbatimElevation: 1470 m; verbatimCoordinateSystem: 35°52’00’’ N, 01°58’07’’ E; **Identification:** identifiedBy: Ourida Kherbouche-Abrous; **Event:** samplingProtocol: pitfall traps; year: 2019; month: 2; day: 1-15
- **Type status:** Other material. **Occurrence:** recordedBy: Amina Saadi; individualCount: 4; sex: 1 male, 3 females; lifeStage: adult; occurrenceID: 6E899FDE-BE0F-5435-B521-4F237A3B03B1; **Taxon:** scientificNameID: urn:lsid:nmbe.ch:spidersp:009777; scientificName: *Centromerussuccinus* (Simon, 1884); kingdom: Animalia; phylum: Arthropoda; class: Arachnida; order: Araneae; family: Linyphiidae; genus: Centromerus; taxonRank: species; taxonomicStatus: accepted; **Location:** higherGeography: North Africa; continent: Africa; country: Algeria; countryCode: DZ; municipality: Theniet El Had; verbatimElevation: 1456 m; verbatimCoordinateSystem: 35°51’18’’ N, 01°59’20’’ E; **Identification:** identifiedBy: Ourida Kherbouche-Abrous; **Event:** samplingProtocol: pitfall traps; year: 2019; month: 5; day: 1-15
- **Type status:** Other material. **Occurrence:** recordedBy: Amina Saadi; individualCount: 2; sex: 2 females; lifeStage: adult; occurrenceID: 8B0F6B08-776C-54E8-B2DB-98E295D8C98E; **Taxon:** scientificNameID: urn:lsid:nmbe.ch:spidersp:009777; scientificName: *Centromerussuccinus* (Simon, 1884); kingdom: Animalia; phylum: Arthropoda; class: Arachnida; order: Araneae; family: Linyphiidae; genus: Centromerus; taxonRank: species; taxonomicStatus: accepted; **Location:** higherGeography: North Africa; continent: Africa; country: Algeria; countryCode: DZ; municipality: Theniet El Had; verbatimElevation: 1474 m; verbatimCoordinateSystem: 35°51’58’’ N, 01°58’06’’E; **Identification:** identifiedBy: Ourida Kherbouche-Abrous; **Event:** samplingProtocol: pitfall traps; year: 2019; month: 6; day: 1-15
- **Type status:** Other material. **Occurrence:** recordedBy: Amina Saadi; individualCount: 2; sex: 2 females; lifeStage: adult; occurrenceID: DCF115B5-60BA-5D2E-9FB8-86CE381B4A36; **Taxon:** scientificNameID: urn:lsid:nmbe.ch:spidersp:009777; scientificName: *Centromerussuccinus* (Simon, 1884); kingdom: Animalia; phylum: Arthropoda; class: Arachnida; order: Araneae; family: Linyphiidae; genus: Centromerus; taxonRank: species; taxonomicStatus: accepted; **Location:** higherGeography: North Africa; continent: Africa; country: Algeria; countryCode: DZ; municipality: Theniet El Had; verbatimElevation: 1474 m; verbatimCoordinateSystem: 35°51’58’’ N, 01°58’06’’E; **Identification:** identifiedBy: Ourida Kherbouche-Abrous; **Event:** samplingProtocol: pitfall traps; year: 2019; month: 7; day: 1-15
- **Type status:** Other material. **Occurrence:** recordedBy: Amina Saadi; individualCount: 1; sex: 1 male; lifeStage: adult; occurrenceID: A512B257-0418-5113-AB92-6D8154860AD8; **Taxon:** scientificNameID: urn:lsid:nmbe.ch:spidersp:009777; scientificName: *Centromerussuccinus* (Simon, 1884); kingdom: Animalia; phylum: Arthropoda; class: Arachnida; order: Araneae; family: Linyphiidae; genus: Centromerus; taxonRank: species; taxonomicStatus: accepted; **Location:** higherGeography: North Africa; continent: Africa; country: Algeria; countryCode: DZ; municipality: Theniet El Had; verbatimElevation: 1474 m; verbatimCoordinateSystem: 35°51’58’’ N, 01°58’06’’E; **Identification:** identifiedBy: Ourida Kherbouche-Abrous; **Event:** samplingProtocol: pitfall traps; year: 2019; month: 10; day: 1-15
- **Type status:** Other material. **Occurrence:** recordedBy: Amina Saadi; individualCount: 11; sex: 11 males; lifeStage: adult; occurrenceID: 6970110D-4A01-5908-83C5-8E3AC2624162; **Taxon:** scientificNameID: urn:lsid:nmbe.ch:spidersp:009777; scientificName: *Centromerussuccinus* (Simon, 1884); kingdom: Animalia; phylum: Arthropoda; class: Arachnida; order: Araneae; family: Linyphiidae; genus: Centromerus; taxonRank: species; taxonomicStatus: accepted; **Location:** higherGeography: North Africa; continent: Africa; country: Algeria; countryCode: DZ; municipality: Theniet El Had; verbatimElevation: 1470 m; verbatimCoordinateSystem: 35°52’00’’ N, 01°58’07’’ E; **Identification:** identifiedBy: Ourida Kherbouche-Abrous; **Event:** samplingProtocol: pitfall traps; year: 2019; month: 10; day: 1-15
- **Type status:** Other material. **Occurrence:** recordedBy: Amina Saadi; individualCount: 1; sex: 1 male; lifeStage: adult; occurrenceID: 2D432921-4E6A-5059-8778-D3E59EFE0E1C; **Taxon:** scientificNameID: urn:lsid:nmbe.ch:spidersp:009777; scientificName: *Centromerussuccinus* (Simon, 1884); kingdom: Animalia; phylum: Arthropoda; class: Arachnida; order: Araneae; family: Linyphiidae; genus: Centromerus; taxonRank: species; taxonomicStatus: accepted; **Location:** higherGeography: North Africa; continent: Africa; country: Algeria; countryCode: DZ; municipality: Theniet El Had; verbatimElevation: 1470 m; verbatimCoordinateSystem: 35°52’00’’ N, 01°58’07’’ E; **Identification:** identifiedBy: Ourida Kherbouche-Abrous; **Event:** samplingProtocol: pitfall traps; year: 2019; month: 11; day: 1-15
- **Type status:** Other material. **Occurrence:** recordedBy: Amina Saadi; individualCount: 3; sex: 3 males; lifeStage: adult; occurrenceID: BFE8050F-92CF-5AF6-AAB0-197C66CE5F85; **Taxon:** scientificNameID: urn:lsid:nmbe.ch:spidersp:009777; scientificName: *Centromerussuccinus* (Simon, 1884); kingdom: Animalia; phylum: Arthropoda; class: Arachnida; order: Araneae; family: Linyphiidae; genus: Centromerus; taxonRank: species; taxonomicStatus: accepted; **Location:** higherGeography: North Africa; continent: Africa; country: Algeria; countryCode: DZ; municipality: Theniet El Had; verbatimElevation: 1474 m; verbatimCoordinateSystem: 35°51’58’’ N, 01°58’06’’E; **Identification:** identifiedBy: Ourida Kherbouche-Abrous; **Event:** samplingProtocol: pitfall traps; year: 2019; month: 11; day: 1-15
- **Type status:** Other material. **Occurrence:** recordedBy: Amina Saadi; individualCount: 1; sex: 1 male; lifeStage: adult; occurrenceID: 4BB55DAE-4943-53AF-83EE-20438EDBAE2B; **Taxon:** scientificNameID: urn:lsid:nmbe.ch:spidersp:009777; scientificName: *Centromerussuccinus* (Simon, 1884); kingdom: Animalia; phylum: Arthropoda; class: Arachnida; order: Araneae; family: Linyphiidae; genus: Centromerus; taxonRank: species; taxonomicStatus: accepted; **Location:** higherGeography: North Africa; continent: Africa; country: Algeria; countryCode: DZ; municipality: Theniet El Had; verbatimElevation: 1448 m; verbatimCoordinateSystem: 35°51’30’’ N, 01°59’11’’ E; **Identification:** identifiedBy: Ourida Kherbouche-Abrous; **Event:** samplingProtocol: pitfall traps; year: 2019; month: 11; day: 1-15
- **Type status:** Other material. **Occurrence:** recordedBy: Amina Saadi; individualCount: 1; sex: 1 male; lifeStage: adult; occurrenceID: 182F604D-89F3-5B61-86BE-7D7C858D269B; **Taxon:** scientificNameID: urn:lsid:nmbe.ch:spidersp:009777; scientificName: *Centromerussuccinus* (Simon, 1884); kingdom: Animalia; phylum: Arthropoda; class: Arachnida; order: Araneae; family: Linyphiidae; genus: Centromerus; taxonRank: species; taxonomicStatus: accepted; **Location:** higherGeography: North Africa; continent: Africa; country: Algeria; countryCode: DZ; municipality: Theniet El Had; verbatimElevation: 1456 m; verbatimCoordinateSystem: 35°51’18’’ N, 01°59’20’’ E; **Identification:** identifiedBy: Ourida Kherbouche-Abrous; **Event:** samplingProtocol: pitfall traps; year: 2019; month: 11; day: 1-15
- **Type status:** Other material. **Occurrence:** recordedBy: Badis Bakhouche; individualCount: 1; sex: 1 male; lifeStage: adult; occurrenceID: 0EE5A1DF-09FA-5445-91BE-3173781650E2; **Taxon:** scientificNameID: urn:lsid:nmbe.ch:spidersp:009777; scientificName: *Centromerussuccinus* (Simon, 1884); kingdom: Animalia; phylum: Arthropoda; class: Arachnida; order: Araneae; family: Linyphiidae; genus: Centromerus; taxonRank: species; taxonomicStatus: accepted; **Location:** higherGeography: North Africa; continent: Africa; country: Algeria; countryCode: DZ; municipality: Theniet El Had; verbatimElevation: 1448 m; verbatimCoordinateSystem: 35°51’30’’ N, 01°59’11’’ E; **Identification:** identifiedBy: Ourida Kherbouche-Abrous; **Event:** samplingProtocol: hand collecting; year: 2019; month: 11; day: 1-15
- **Type status:** Other material. **Occurrence:** recordedBy: Amina Saadi; individualCount: 10; sex: 7 males, 3 females; lifeStage: adult; occurrenceID: 1D2137F4-6C1C-50A6-AA0E-9382E9047BC6; **Taxon:** scientificNameID: urn:lsid:nmbe.ch:spidersp:009777; scientificName: *Centromerussuccinus* (Simon, 1884); kingdom: Animalia; phylum: Arthropoda; class: Arachnida; order: Araneae; family: Linyphiidae; genus: Centromerus; taxonRank: species; taxonomicStatus: accepted; **Location:** higherGeography: North Africa; continent: Africa; country: Algeria; countryCode: DZ; municipality: Theniet El Had; verbatimElevation: 1470 m; verbatimCoordinateSystem: 35°52’00’’ N, 01°58’07’’ E; **Identification:** identifiedBy: Ourida Kherbouche-Abrous; **Event:** samplingProtocol: pitfall traps; year: 2019; month: 12; day: 16-31
- **Type status:** Other material. **Occurrence:** recordedBy: Amina Saadi; individualCount: 4; sex: 1 male, 4 females; lifeStage: adult; occurrenceID: BACA3584-F6A9-5D24-8A00-0CEEF97AFD7B; **Taxon:** scientificNameID: urn:lsid:nmbe.ch:spidersp:009777; scientificName: *Centromerussuccinus* (Simon, 1884); kingdom: Animalia; phylum: Arthropoda; class: Arachnida; order: Araneae; family: Linyphiidae; genus: Centromerus; taxonRank: species; taxonomicStatus: accepted; **Location:** higherGeography: North Africa; continent: Africa; country: Algeria; countryCode: DZ; municipality: Theniet El Had; verbatimElevation: 1474 m; verbatimCoordinateSystem: 35°51’58’’ N, 01°58’06’’E; **Identification:** identifiedBy: Ourida Kherbouche-Abrous; **Event:** samplingProtocol: pitfall traps; year: 2019; month: 12; day: 16-31

#### Distribution

**Algerian distribution**: North part of the country (Bosmans 1986, Bosmans 2006b).

**Global distribution**: Portugal, Spain, France, Switzerland, Italy, Algeria (World Spider Catalog 2024).

### Diplocephalus graecus

(O. Pickard-Cambridge, 1873)

F214396D-849D-5FD1-8C27-AE230965C7D3

#### Materials

- **Type status:** Other material. **Occurrence:** recordedBy: Badis Bakhouche; individualCount: 2; sex: 2 males; lifeStage: adult; occurrenceID: 9D606C95-8A7F-55A0-B0AE-216569B54DD4; **Taxon:** scientificNameID: urn:lsid:nmbe.ch:spidersp:010058; scientificName: *Diplocephalusgraecus* (O. Pickard-Cambridge, 1873); kingdom: Animalia; phylum: Arthropoda; class: Arachnida; order: Araneae; family: Linyphiidae; genus: Diplocephalus; taxonRank: species; taxonomicStatus: accepted; **Location:** higherGeography: North Africa; continent: Africa; country: Algeria; countryCode: DZ; municipality: Theniet El Had; verbatimElevation: 1474 m; verbatimCoordinateSystem: 35°51’58’’ N, 01°58’06’’E; **Identification:** identifiedBy: Ourida Kherbouche-Abrous; **Event:** samplingProtocol: hand collecting; year: 2019; month: 11; day: 1-15
- **Type status:** Other material. **Occurrence:** recordedBy: Amina Saadi; individualCount: 3; sex: 3 males; lifeStage: adult; occurrenceID: 10EDFEAE-3FD2-5DF2-993C-0F87747DAB5C; **Taxon:** scientificNameID: urn:lsid:nmbe.ch:spidersp:010058; scientificName: *Diplocephalusgraecus* (O. Pickard-Cambridge, 1873); kingdom: Animalia; phylum: Arthropoda; class: Arachnida; order: Araneae; family: Linyphiidae; genus: Diplocephalus; taxonRank: species; taxonomicStatus: accepted; **Location:** higherGeography: North Africa; continent: Africa; country: Algeria; countryCode: DZ; municipality: Theniet El Had; verbatimElevation: 1448 m; verbatimCoordinateSystem: 35°51’30’’ N, 01°59’11’’ E; **Identification:** identifiedBy: Ourida Kherbouche-Abrous; **Event:** samplingProtocol: pitfall traps; year: 2019; month: 12; day: 16-31
- **Type status:** Other material. **Occurrence:** recordedBy: Amina Saadi; individualCount: 9; sex: 7 males, 2 females; lifeStage: adult; occurrenceID: 163EE06D-D883-5662-9E1C-F8154B84C780; **Taxon:** scientificNameID: urn:lsid:nmbe.ch:spidersp:010058; scientificName: *Diplocephalusgraecus* (O. Pickard-Cambridge, 1873); kingdom: Animalia; phylum: Arthropoda; class: Arachnida; order: Araneae; family: Linyphiidae; genus: Diplocephalus; taxonRank: species; taxonomicStatus: accepted; **Location:** higherGeography: North Africa; continent: Africa; country: Algeria; countryCode: DZ; municipality: Theniet El Had; verbatimElevation: 1470 m; verbatimCoordinateSystem: 35°52’00’’ N, 01°58’07’’ E; **Identification:** identifiedBy: Ourida Kherbouche-Abrous; **Event:** samplingProtocol: pitfall traps; year: 2019; month: 12; day: 16-31
- **Type status:** Other material. **Occurrence:** recordedBy: Amina Saadi; individualCount: 1; sex: 1 male; lifeStage: adult; occurrenceID: 02C4597B-824C-5195-BFA3-E3F810047B1E; **Taxon:** scientificNameID: urn:lsid:nmbe.ch:spidersp:010058; scientificName: *Diplocephalusgraecus* (O. Pickard-Cambridge, 1873); kingdom: Animalia; phylum: Arthropoda; class: Arachnida; order: Araneae; family: Linyphiidae; genus: Diplocephalus; taxonRank: species; taxonomicStatus: accepted; **Location:** higherGeography: North Africa; continent: Africa; country: Algeria; countryCode: DZ; municipality: Theniet El Had; verbatimElevation: 1456 m; verbatimCoordinateSystem: 35°51’18’’ N, 01°59’20’’ E; **Identification:** identifiedBy: Ourida Kherbouche-Abrous; **Event:** samplingProtocol: pitfall traps; year: 2019; month: 12; day: 16-31

#### Distribution

**Algerian distribution**: The north part of the country (Simon 1884, Bosmans 1996, Bouseksou et al. 2015).

**Global distribution**: Madeira, Europe, North Africa, Turkey, Israel (World Spider Catalog 2024).

### Gonatium occidentale

Simon, 1918

C0BC665D-52BF-5899-B1A4-E63980F05901

#### Materials

- **Type status:** Other material. **Occurrence:** recordedBy: Badis Bakhouche; individualCount: 1; sex: 1 female; lifeStage: adult; occurrenceID: EB000231-3AA5-5181-9F77-035A24E7DD6D; **Taxon:** scientificNameID: urn:lsid:nmbe.ch:spidersp:010666; scientificName: *Gonatiumoccidentale* Simon, 1918; kingdom: Animalia; phylum: Arthropoda; class: Arachnida; order: Araneae; family: Linyphiidae; genus: Gonatium; taxonRank: species; taxonomicStatus: accepted; **Location:** higherGeography: North Africa; continent: Africa; country: Algeria; countryCode: DZ; municipality: Theniet El Had; verbatimElevation: 1449 m; verbatimCoordinateSystem: 35°51’34’’ N, 01°58’50’’ E; **Identification:** identifiedBy: Ourida Kherbouche-Abrous; **Event:** samplingProtocol: hand collecting; year: 2019; month: 1; day: 1-15
- **Type status:** Other material. **Occurrence:** recordedBy: Badis Bakhouche; individualCount: 1; sex: 1 female; lifeStage: adult; occurrenceID: 34B77F43-D7D4-5BCE-A07A-A9ABD724178C; **Taxon:** scientificNameID: urn:lsid:nmbe.ch:spidersp:010666; scientificName: *Gonatiumoccidentale* Simon, 1918; kingdom: Animalia; phylum: Arthropoda; class: Arachnida; order: Araneae; family: Linyphiidae; genus: Gonatium; taxonRank: species; taxonomicStatus: accepted; **Location:** higherGeography: North Africa; continent: Africa; country: Algeria; countryCode: DZ; municipality: Theniet El Had; verbatimElevation: 1474 m; verbatimCoordinateSystem: 35°51’58’’ N, 01°58’06’’E; **Identification:** identifiedBy: Ourida Kherbouche-Abrous; **Event:** samplingProtocol: hand collecting; year: 2019; month: 1; day: 1-15
- **Type status:** Other material. **Occurrence:** recordedBy: Badis Bakhouche; individualCount: 1; sex: 1 female; lifeStage: adult; occurrenceID: FD31D90E-EE0C-5061-9A64-06724DC20723; **Taxon:** scientificNameID: urn:lsid:nmbe.ch:spidersp:010666; scientificName: *Gonatiumoccidentale* Simon, 1918; kingdom: Animalia; phylum: Arthropoda; class: Arachnida; order: Araneae; family: Linyphiidae; genus: Gonatium; taxonRank: species; taxonomicStatus: accepted; **Location:** higherGeography: North Africa; continent: Africa; country: Algeria; countryCode: DZ; municipality: Theniet El Had; verbatimElevation: 1474 m; verbatimCoordinateSystem: 35°51’58’’ N, 01°58’06’’E; **Identification:** identifiedBy: Ourida Kherbouche-Abrous; **Event:** samplingProtocol: hand collecting; year: 2019; month: 3; day: 1-15
- **Type status:** Other material. **Occurrence:** recordedBy: Amina Saadi; individualCount: 1; sex: 1 male; lifeStage: adult; occurrenceID: 709B9417-B1DB-5300-B626-45AC332E58C1; **Taxon:** scientificNameID: urn:lsid:nmbe.ch:spidersp:010666; scientificName: *Gonatiumoccidentale* Simon, 1918; kingdom: Animalia; phylum: Arthropoda; class: Arachnida; order: Araneae; family: Linyphiidae; genus: Gonatium; taxonRank: species; taxonomicStatus: accepted; **Location:** higherGeography: North Africa; continent: Africa; country: Algeria; countryCode: DZ; municipality: Theniet El Had; verbatimElevation: 1456 m; verbatimCoordinateSystem: 35°51’18’’ N, 01°59’20’’ E; **Identification:** identifiedBy: Ourida Kherbouche-Abrous; **Event:** samplingProtocol: pitfall traps; year: 2019; month: 10; day: 1-15

#### Distribution

**Algerian distribution**: North part of the country (Bosmans 2007).

**Global distribution**: Spain, France, Morocco, Algeria, Israel (World Spider Catalog 2024).

### Improphantes decolor

(Westring, 1861)

886ABBA6-D675-54E9-82AF-A866AB0B6D25

#### Materials

- **Type status:** Other material. **Occurrence:** recordedBy: Amina Saadi; individualCount: 2; sex: 1 male, 1 female; lifeStage: adult; occurrenceID: EBBAC5FB-5049-5307-9DD7-8356669D968F; **Taxon:** scientificNameID: urn:lsid:nmbe.ch:spidersp:010894; scientificName: *Improphantesdecolor* (Westring, 1861); kingdom: Animalia; phylum: Arthropoda; class: Arachnida; order: Araneae; family: Linyphiidae; genus: Improphantes; taxonRank: species; taxonomicStatus: accepted; **Location:** higherGeography: North Africa; continent: Africa; country: Algeria; countryCode: DZ; municipality: Theniet El Had; verbatimElevation: 1456 m; verbatimCoordinateSystem: 35°51’18’’ N, 01°59’20’’ E; **Identification:** identifiedBy: Ourida Kherbouche-Abrous; **Event:** samplingProtocol: pitfall traps; year: 2019; month: 1; day: 1-15
- **Type status:** Other material. **Occurrence:** recordedBy: Badis Bakhouche; individualCount: 2; sex: 2 males; lifeStage: adult; occurrenceID: C8BD1983-B76B-5122-A3C5-008D4EDA4193; **Taxon:** scientificNameID: urn:lsid:nmbe.ch:spidersp:010894; scientificName: *Improphantesdecolor* (Westring, 1861); kingdom: Animalia; phylum: Arthropoda; class: Arachnida; order: Araneae; family: Linyphiidae; genus: Improphantes; taxonRank: species; taxonomicStatus: accepted; **Location:** higherGeography: North Africa; continent: Africa; country: Algeria; countryCode: DZ; municipality: Theniet El Had; verbatimElevation: 1456 m; verbatimCoordinateSystem: 35°51’18’’ N, 01°59’20’’ E; **Identification:** identifiedBy: Ourida Kherbouche-Abrous; **Event:** samplingProtocol: hand collecting; year: 2019; month: 4; day: 1-15
- **Type status:** Other material. **Occurrence:** recordedBy: Badis Bakhouche; individualCount: 3; sex: 3 females; lifeStage: adult; occurrenceID: 8342A36F-7C79-5E16-83CE-E159FF4758B7; **Taxon:** scientificNameID: urn:lsid:nmbe.ch:spidersp:010894; scientificName: *Improphantesdecolor* (Westring, 1861); kingdom: Animalia; phylum: Arthropoda; class: Arachnida; order: Araneae; family: Linyphiidae; genus: Improphantes; taxonRank: species; taxonomicStatus: accepted; **Location:** higherGeography: North Africa; continent: Africa; country: Algeria; countryCode: DZ; municipality: Theniet El Had; verbatimElevation: 1448 m; verbatimCoordinateSystem: 35°51’30’’ N, 01°59’11’’ E; **Identification:** identifiedBy: Ourida Kherbouche-Abrous; **Event:** samplingProtocol: hand collecting; year: 2019; month: 4; day: 1-15
- **Type status:** Other material. **Occurrence:** recordedBy: Amina Saadi; individualCount: 1; sex: 1 male; lifeStage: adult; occurrenceID: 984C114F-23FD-5690-AE6A-013741B65516; **Taxon:** scientificNameID: urn:lsid:nmbe.ch:spidersp:010894; scientificName: *Improphantesdecolor* (Westring 1861); kingdom: Animalia; phylum: Arthropoda; class: Arachnida; order: Araneae; family: Linyphiidae; genus: Improphantes; taxonRank: species; taxonomicStatus: accepted; **Location:** higherGeography: North Africa; continent: Africa; country: Algeria; countryCode: DZ; municipality: Theniet El Had; verbatimElevation: 1448 m; verbatimCoordinateSystem: 35°51’30’’ N, 01°59’11’’ E; **Identification:** identifiedBy: Ourida Kherbouche-Abrous; **Event:** samplingProtocol: pitfall traps; year: 2019; month: 4; day: 1-15
- **Type status:** Other material. **Occurrence:** recordedBy: Amina Saadi; individualCount: 1; sex: 1 female; lifeStage: adult; occurrenceID: 9C1C7574-92A4-51A3-9270-6F05EAD2EA43; **Taxon:** scientificNameID: urn:lsid:nmbe.ch:spidersp:010894; scientificName: *Improphantesdecolor* (Westring, 1861); kingdom: Animalia; phylum: Arthropoda; class: Arachnida; order: Araneae; family: Linyphiidae; genus: Improphantes; taxonRank: species; taxonomicStatus: accepted; **Location:** higherGeography: North Africa; continent: Africa; country: Algeria; countryCode: DZ; municipality: Theniet El Had; verbatimElevation: 1448 m; verbatimCoordinateSystem: 35°51’30’’ N, 01°59’11’’ E; **Identification:** identifiedBy: Ourida Kherbouche-Abrous; **Event:** samplingProtocol: pitfall traps; year: 2019; month: 5; day: 1-15
- **Type status:** Other material. **Occurrence:** recordedBy: Amina Saadi; individualCount: 2; sex: 2 females; lifeStage: adult; occurrenceID: 1D9FBB4C-1FC6-5474-B1BA-F1D48E815FAD; **Taxon:** scientificNameID: urn:lsid:nmbe.ch:spidersp:010894; scientificName: *Improphantesdecolor* (Westring,1861); kingdom: Animalia; phylum: Arthropoda; class: Arachnida; order: Araneae; family: Linyphiidae; genus: Improphantes; taxonRank: species; taxonomicStatus: accepted; **Location:** higherGeography: North Africa; continent: Africa; country: Algeria; countryCode: DZ; municipality: Theniet El Had; verbatimElevation: 1456 m; verbatimCoordinateSystem: 35°51’18’’ N, 01°59’20’’ E; **Identification:** identifiedBy: Ourida Kherbouche-Abrous; **Event:** samplingProtocol: pitfall traps; year: 2019; month: 5; day: 1-15
- **Type status:** Other material. **Occurrence:** recordedBy: Amina Saadi; individualCount: 1; sex: 1 female; lifeStage: adult; occurrenceID: E4421A00-36C8-5004-9E6C-780F4CC11420; **Taxon:** scientificNameID: urn:lsid:nmbe.ch:spidersp:010894; scientificName: *Improphantesdecolor* (Westring, 1861); kingdom: Animalia; phylum: Arthropoda; class: Arachnida; order: Araneae; family: Linyphiidae; genus: Improphantes; taxonRank: species; taxonomicStatus: accepted; **Location:** higherGeography: North Africa; continent: Africa; country: Algeria; countryCode: DZ; municipality: Theniet El Had; verbatimElevation: 1456 m; verbatimCoordinateSystem: 35°51’18’’ N, 01°59’20’’ E; **Identification:** identifiedBy: Ourida Kherbouche-Abrous; **Event:** samplingProtocol: pitfall traps; year: 2019; month: 10; day: 1-15
- **Type status:** Other material. **Occurrence:** recordedBy: Amina Saadi; individualCount: 4; sex: 4 females; lifeStage: adult; occurrenceID: 5F605DBD-196D-5FAA-8E9C-B9C048CAD1A5; **Taxon:** scientificNameID: urn:lsid:nmbe.ch:spidersp:010894; scientificName: *Improphantesdecolor* (Westring, 1861); kingdom: Animalia; phylum: Arthropoda; class: Arachnida; order: Araneae; family: Linyphiidae; genus: Improphantes; taxonRank: species; taxonomicStatus: accepted; **Location:** higherGeography: North Africa; continent: Africa; country: Algeria; countryCode: DZ; municipality: Theniet El Had; verbatimElevation: 1470 m; verbatimCoordinateSystem: 35°52’00’’ N, 01°58’07’’ E; **Identification:** identifiedBy: Ourida Kherbouche-Abrous; **Event:** samplingProtocol: pitfall traps; year: 2019; month: 10; day: 1-15
- **Type status:** Other material. **Occurrence:** recordedBy: Amina Saadi; individualCount: 3; sex: 3 females; lifeStage: adult; occurrenceID: 130C4E3E-BB8B-53BD-9DA0-B19BCBDCD247; **Taxon:** scientificNameID: urn:lsid:nmbe.ch:spidersp:010894; scientificName: *Improphantesdecolor* (Westring, 1861); kingdom: Animalia; phylum: Arthropoda; class: Arachnida; order: Araneae; family: Linyphiidae; genus: Improphantes; taxonRank: species; taxonomicStatus: accepted; **Location:** higherGeography: North Africa; continent: Africa; country: Algeria; countryCode: DZ; municipality: Theniet El Had; verbatimElevation: 1470 m; verbatimCoordinateSystem: 35°52’00’’ N, 01°58’07’’ E; **Identification:** identifiedBy: Ourida Kherbouche-Abrous; **Event:** samplingProtocol: pitfall traps; year: 2019; month: 11; day: 1-15
- **Type status:** Other material. **Occurrence:** recordedBy: Amina Saadi; individualCount: 1; sex: 1 female; lifeStage: adult; occurrenceID: 8A316D79-DA8E-549A-BB31-36074A17472B; **Taxon:** scientificNameID: urn:lsid:nmbe.ch:spidersp:010894; scientificName: *Improphantesdecolor* (Westring, 1861); kingdom: Animalia; phylum: Arthropoda; class: Arachnida; order: Araneae; family: Linyphiidae; genus: Improphantes; taxonRank: species; taxonomicStatus: accepted; **Location:** higherGeography: North Africa; continent: Africa; country: Algeria; countryCode: DZ; municipality: Theniet El Had; verbatimElevation: 1456 m; verbatimCoordinateSystem: 35°51’18’’ N, 01°59’20’’ E; **Identification:** identifiedBy: Ourida Kherbouche-Abrous; **Event:** samplingProtocol: pitfall traps; year: 2019; month: 11; day: 1-15
- **Type status:** Other material. **Occurrence:** recordedBy: Amina Saadi; individualCount: 1; sex: 1 female; lifeStage: adult; occurrenceID: EC70908B-5DFC-58B7-B0CE-E10C9CA062C4; **Taxon:** scientificNameID: urn:lsid:nmbe.ch:spidersp:010894; scientificName: *Improphantesdecolor* (Westring, 1861); kingdom: Animalia; phylum: Arthropoda; class: Arachnida; order: Araneae; family: Linyphiidae; genus: Improphantes; taxonRank: species; taxonomicStatus: accepted; **Location:** higherGeography: North Africa; continent: Africa; country: Algeria; countryCode: DZ; municipality: Theniet El Had; verbatimElevation: 1474 m; verbatimCoordinateSystem: 35°51’58’’ N, 01°58’06’’ E; **Identification:** identifiedBy: Ourida Kherbouche-Abrous; **Event:** samplingProtocol: pitfall traps; year: 2019; month: 11; day: 1-15
- **Type status:** Other material. **Occurrence:** recordedBy: Amina Saadi; individualCount: 1; sex: 1 male; lifeStage: adult; occurrenceID: 6A36716B-6A82-5C98-8B9D-48CB6EC04656; **Taxon:** scientificNameID: urn:lsid:nmbe.ch:spidersp:010894; scientificName: *Improphantesdecolor* (Westring, 1861); kingdom: Animalia; phylum: Arthropoda; class: Arachnida; order: Araneae; family: Linyphiidae; genus: Improphantes; taxonRank: species; taxonomicStatus: accepted; **Location:** higherGeography: North Africa; continent: Africa; country: Algeria; countryCode: DZ; municipality: Theniet El Had; verbatimElevation: 1474 m; verbatimCoordinateSystem: 35°51’58’’ N, 01°58’06’’ E; **Identification:** identifiedBy: Ourida Kherbouche-Abrous; **Event:** samplingProtocol: pitfall traps; year: 2019; month: 12; day: 16-31
- **Type status:** Other material. **Occurrence:** recordedBy: Amina Saadi; individualCount: 3; sex: 3 females; lifeStage: adult; occurrenceID: 376C7A01-2129-58BD-8CBC-D670774B0F81; **Taxon:** scientificNameID: urn:lsid:nmbe.ch:spidersp:010894; scientificName: *Improphantesdecolor* (Westring, 1861); kingdom: Animalia; phylum: Arthropoda; class: Arachnida; order: Araneae; family: Linyphiidae; genus: Improphantes; taxonRank: species; taxonomicStatus: accepted; **Location:** higherGeography: North Africa; continent: Africa; country: Algeria; countryCode: DZ; municipality: Theniet El Had; verbatimElevation: 1470 m; verbatimCoordinateSystem: 35°52’00’’ N, 01°58’07’’ E; **Identification:** identifiedBy: Ourida Kherbouche-Abrous; **Event:** samplingProtocol: pitfall traps; year: 2019; month: 12; day: 16-31
- **Type status:** Other material. **Occurrence:** recordedBy: Badis Bakhouche; individualCount: 2; sex: 1 male, 1 females; lifeStage: adult; occurrenceID: 8A2E7989-C9C5-5794-A1B4-8E6B61B394A9; **Taxon:** scientificNameID: urn:lsid:nmbe.ch:spidersp:010894; scientificName: *Improphantesdecolor* (Westring, 1861); kingdom: Animalia; phylum: Arthropoda; class: Arachnida; order: Araneae; family: Linyphiidae; genus: Improphantes; taxonRank: species; taxonomicStatus: accepted; **Location:** higherGeography: North Africa; continent: Africa; country: Algeria; countryCode: DZ; municipality: Theniet El Had; verbatimElevation: 1470 m; verbatimCoordinateSystem: 35°52’00’’ N, 01°58’07’’ E; **Identification:** identifiedBy: Ourida Kherbouche-Abrous; **Event:** samplingProtocol: hand collecting; year: 2019; month: 12; day: 16-31

#### Distribution

**Algerian distribution**: The north part of the country (Bosmans 2006a, Bouseksou et al. 2015, Touchi et al. 2018).

**Global distribution**: Europe, North Africa (World Spider Catalog 2024).

### Lessertia barbara

(Simon, 1884)

79AA8193-91AB-5CF6-A1E3-03366ACA6AA3

#### Materials

- **Type status:** Other material. **Occurrence:** recordedBy: Amina Saadi; individualCount: 1; sex: 1 male; lifeStage: adult; occurrenceID: 66FA3ECD-5E6B-557E-BEC3-00993DC76024; **Taxon:** scientificNameID: urn:lsid:nmbe.ch:spidersp:011342; scientificName: *Lessertiabarbara* (Simon, 1884); kingdom: Animalia ; phylum: Arthropoda ; class: Arachnida ; order: Araneae ; family: Linyphiidae ; genus: Lessertia ; taxonRank: species; taxonomicStatus: accepted; **Location:** higherGeography: North Africa; continent: Africa; country: Algeria; countryCode: DZ; municipality: Theniet El Had; verbatimElevation: 1456 m; verbatimCoordinateSystem: 35°51’18’’ N, 01°59’20’’ E; **Identification:** identifiedBy: Ourida Kherbouche-Abrous; **Event:** samplingProtocol: pitfall traps; year: 2019; month: 10; day: 1-15

#### Distribution

**Algerian distribution**: The north part of the country (Bosmans 2007).

**Global distribution**: Spain, Morocco, Algeria, Italy (Sicily) (World Spider Catalog 2024).

### Mecopisthes monticola

Bosmans, 1994

E50EDA00-B17E-5560-8C0D-0FEB560E9CBE

#### Materials

- **Type status:** Other material. **Occurrence:** recordedBy: Amina Saadi; individualCount: 2; sex: 2 males; lifeStage: adult; occurrenceID: 85BDF751-59EA-5899-BB62-182B0A7C1BE2; **Taxon:** scientificNameID: urn:lsid:nmbe.ch:spidersp:011546; scientificName: *Mecopisthesmonticola* Bosmans, 1994; kingdom: Animalia; phylum: Arthropoda; class: Arachnida; order: Araneae; family: Linyphiidae; genus: Mecopisthes; taxonRank: species; taxonomicStatus: accepted; **Location:** higherGeography: North Africa; continent: Africa; country: Algeria; countryCode: DZ; municipality: Theniet El Had; verbatimElevation: 1470 m; verbatimCoordinateSystem: 35°52’00’’ N, 01°58’07’’ E; **Identification:** identifiedBy: Ourida Kherbouche-Abrous; **Event:** samplingProtocol: pitfall traps; year: 2019; month: 12; day: 16-31

#### Distribution

**Algerian distribution**: North part, from Tiaret in the west to Setif in the east (Bosmans and Chergui 1993, Bouseksou et al. 2015, Mansouri et al. 2019).

**Global distribution**: Algeria (World Spider Catalog 2024).

### Nematogmus sanguinolentus

(Walckenaer, 1841)

287BFF99-927C-5A6E-9A46-BE2D93557AE1

#### Materials

- **Type status:** Other material. **Occurrence:** recordedBy: Badis Bakhouche; individualCount: 2; sex: 2 females; lifeStage: adult; occurrenceID: 207B2552-B53A-5442-A81E-DDC2A152816F; **Taxon:** scientificNameID: urn:lsid:nmbe.ch:spidersp:011936; scientificName: *Nematogmussanguinolentus* (Walckenaer, 1841); kingdom: Animalia; phylum: Arthropoda; class: Arachnida; order: Araneae; family: Linyphiidae; genus: Nematogmus; **Location:** higherGeography: North Africa; continent: Africa; country: Algeria; countryCode: DZ; municipality: Theniet El Had; verbatimElevation: 1449 m; verbatimCoordinateSystem: 35°51’34’’ N, 01°58’50’’ E; **Identification:** identifiedBy: Ourida Kherbouche-Abrous; **Event:** samplingProtocol: hand collecting; year: 2019; month: 12; day: 16-31

#### Distribution

**Algerian distribution**: after Simon (1884) without precise locality.

**Global distribution**: Europe, North Africa, Caucasus, Russia (Europe to Far East), China, Korea, Japan (World Spider Catalog 2024).

##### Comment

This is the second record of the species in Algeria since that of Simon (1884).

### Palliduphantes labilis

(Simon, 1913)

4F757E40-A869-515A-B680-5199D6AFE6D0

#### Materials

- **Type status:** Other material. **Occurrence:** recordedBy: Amina Saadi; individualCount: 1; sex: 1 male; lifeStage: adult; occurrenceID: 5973B2AD-ADE8-57B0-86B8-53AF03509401; **Taxon:** scientificNameID: urn:lsid:nmbe.ch:spidersp:011205; scientificName: *Palliduphanteslabilis* (Simon, 1913); kingdom: Animalia; phylum: Arthropoda; class: Arachnida; order: Araneae; family: Linyphiidae; genus: Palliduphantes; taxonRank: species; taxonomicStatus: accepted; **Location:** higherGeography: North Africa; continent: Africa; country: Algeria; countryCode: DZ; municipality: Theniet El Had; verbatimElevation: 1474 m; verbatimCoordinateSystem: 35°51’58’’ N, 01°58’06’’E; **Identification:** identifiedBy: Ourida Kherbouche-Abrous; **Event:** samplingProtocol: pitfall traps; year: 2019; month: 1; day: 1-15
- **Type status:** Other material. **Occurrence:** recordedBy: Badis Bakhouche; individualCount: 2; sex: 1 male, 1 female; lifeStage: adult; occurrenceID: D0DE41B4-3369-554D-A1D7-A7C2F34FB908; **Taxon:** scientificNameID: urn:lsid:nmbe.ch:spidersp:011205; scientificName: *Palliduphanteslabilis* (Simon, 1913); kingdom: Animalia; phylum: Arthropoda; class: Arachnida; order: Araneae; family: Linyphiidae; genus: Palliduphantes; taxonRank: species; taxonomicStatus: accepted; **Location:** higherGeography: North Africa; continent: Africa; country: Algeria; countryCode: DZ; municipality: Theniet El Had; verbatimElevation: 1474 m; verbatimCoordinateSystem: 35°51’58’’ N, 01°58’06’’E; **Identification:** identifiedBy: Ourida Kherbouche-Abrous; **Event:** samplingProtocol: hand collecting; year: 2019; month: 1; day: 1-15
- **Type status:** Other material. **Occurrence:** recordedBy: Amina Saadi; individualCount: 1; sex: 1 female; lifeStage: adult; occurrenceID: E8E64F15-E3DC-5454-8B6F-79E65E807CEE; **Taxon:** scientificNameID: urn:lsid:nmbe.ch:spidersp:011205; scientificName: *Palliduphanteslabilis* (Simon, 1913); kingdom: Animalia; phylum: Arthropoda; class: Arachnida; order: Araneae; family: Linyphiidae; genus: Palliduphantes; taxonRank: species; taxonomicStatus: accepted; **Location:** higherGeography: North Africa; continent: Africa; country: Algeria; countryCode: DZ; municipality: Theniet El Had; verbatimElevation: 1470 m; verbatimCoordinateSystem: 35°52’00’’ N, 01°58’07’’ E; **Identification:** identifiedBy: Ourida Kherbouche-Abrous; **Event:** samplingProtocol: pitfall traps; year: 2019; month: 2; day: 1-15
- **Type status:** Other material. **Occurrence:** recordedBy: Amina Saadi; individualCount: 2; sex: 2 females; lifeStage: adult; occurrenceID: 91F858F3-5DCB-5E9C-B353-3E48F2CF2A2A; **Taxon:** scientificNameID: urn:lsid:nmbe.ch:spidersp:011205; scientificName: *Palliduphanteslabilis* (Simon, 1913); kingdom: Animalia; phylum: Arthropoda; class: Arachnida; order: Araneae; family: Linyphiidae; genus: Palliduphantes; taxonRank: species; taxonomicStatus: accepted; **Location:** higherGeography: North Africa; continent: Africa; country: Algeria; countryCode: DZ; municipality: Theniet El Had; verbatimElevation: 1474 m; verbatimCoordinateSystem: 35°51’58’’ N, 01°58’06’’E; **Identification:** identifiedBy: Ourida Kherbouche-Abrous; **Event:** samplingProtocol: pitfall traps; year: 2019; month: 2; day: 1-15
- **Type status:** Other material. **Occurrence:** recordedBy: Amina Saadi; individualCount: 1; sex: 1male; lifeStage: adult; occurrenceID: D750ACE5-39B4-5C23-B56F-6DF7AB3EFE23; **Taxon:** scientificNameID: urn:lsid:nmbe.ch:spidersp:011205; scientificName: *Palliduphanteslabilis* (Simon, 1913); kingdom: Animalia; phylum: Arthropoda; class: Arachnida; order: Araneae; family: Linyphiidae; genus: Palliduphantes; taxonRank: species; taxonomicStatus: accepted; **Location:** higherGeography: North Africa; continent: Africa; country: Algeria; countryCode: DZ; municipality: Theniet El Had; verbatimElevation: 1449 m; verbatimCoordinateSystem: 35°51’34’’ N, 01°58’50’’ E; **Identification:** identifiedBy: Ourida Kherbouche-Abrous; **Event:** samplingProtocol: pitfall traps; year: 2019; month: 2; day: 1-15
- **Type status:** Other material. **Occurrence:** recordedBy: Badis Bakhouche; individualCount: 1; sex: 1 female; lifeStage: adult; occurrenceID: 2C6B7D3F-8CAF-511C-90FA-5946BF5A9499; **Taxon:** scientificNameID: urn:lsid:nmbe.ch:spidersp:011205; scientificName: *Palliduphanteslabilis* (Simon, 1913); kingdom: Animalia; phylum: Arthropoda; class: Arachnida; order: Araneae; family: Linyphiidae; genus: Palliduphantes; taxonRank: species; taxonomicStatus: accepted; **Location:** higherGeography: North Africa; continent: Africa; country: Algeria; countryCode: DZ; municipality: Theniet El Had; verbatimElevation: 1449 m; verbatimCoordinateSystem: 35°51'34’’ N, 01°58’50’’E; **Identification:** identifiedBy: Ourida Kherbouche-Abrous; **Event:** samplingProtocol: hand collecting; year: 2019; month: 3; day: 1-15
- **Type status:** Other material. **Occurrence:** recordedBy: Amina Saadi; individualCount: 1; sex: 1 female; lifeStage: adult; occurrenceID: FE1BCEAF-8FEC-5214-B67A-0FF65E9E5952; **Taxon:** scientificNameID: urn:lsid:nmbe.ch:spidersp:011205; scientificName: *Palliduphanteslabilis* (Simon, 1913); kingdom: Animalia; phylum: Arthropoda; class: Arachnida; order: Araneae; family: Linyphiidae; genus: Palliduphantes; taxonRank: species; taxonomicStatus: accepted; **Location:** higherGeography: North Africa; continent: Africa; country: Algeria; countryCode: DZ; municipality: Theniet El Had; verbatimElevation: 1448 m; verbatimCoordinateSystem: 35°51’30’’ N, 01°59’11’’E; **Identification:** identifiedBy: Ourida Kherbouche-Abrous; **Event:** samplingProtocol: pitfall traps; year: 2019; month: 4; day: 1-15
- **Type status:** Other material. **Occurrence:** recordedBy: Amina Saadi; individualCount: 2; sex: 1 male, 1 female; lifeStage: adult; occurrenceID: 2664854D-AF10-5952-A05F-479B330C85AD; **Taxon:** scientificNameID: urn:lsid:nmbe.ch:spidersp:011205; scientificName: *Palliduphanteslabilis* (Simon, 1913); kingdom: Animalia; phylum: Arthropoda; class: Arachnida; order: Araneae; family: Linyphiidae; genus: Palliduphantes; taxonRank: species; taxonomicStatus: accepted; **Location:** higherGeography: North Africa; continent: Africa; country: Algeria; countryCode: DZ; municipality: Theniet El Had; verbatimElevation: 1449 m; verbatimCoordinateSystem: 35°51'34’’ N, 01°58’50’’E; **Identification:** identifiedBy: Ourida Kherbouche-Abrous; **Event:** samplingProtocol: pitfall traps; year: 2019; month: 4; day: 1-15
- **Type status:** Other material. **Occurrence:** recordedBy: Badis Bakhouche; individualCount: 1; sex: 1 female; lifeStage: adult; occurrenceID: 583568A0-9957-57D5-9A04-869E685E1077; **Taxon:** scientificNameID: urn:lsid:nmbe.ch:spidersp:011205; scientificName: *Palliduphanteslabilis* (Simon, 1913); kingdom: Animalia; phylum: Arthropoda; class: Arachnida; order: Araneae; family: Linyphiidae; genus: Palliduphantes; taxonRank: species; taxonomicStatus: accepted; **Location:** higherGeography: North Africa; continent: Africa; country: Algeria; countryCode: DZ; municipality: Theniet El Had; verbatimElevation: 1449 m; verbatimCoordinateSystem: 35°51'34’’ N, 01°58’50’’E; **Identification:** identifiedBy: Ourida Kherbouche-Abrous; **Event:** samplingProtocol: hand collecting; year: 2019; month: 4; day: 1-15
- **Type status:** Other material. **Occurrence:** recordedBy: Amina Saadi; individualCount: 1; sex: 1 female; lifeStage: adult; occurrenceID: D0C89473-7CD6-57CB-94B9-66BE6FF3258F; **Taxon:** scientificNameID: urn:lsid:nmbe.ch:spidersp:011205; scientificName: *Palliduphanteslabilis* (Simon, 1913); kingdom: Animalia; phylum: Arthropoda; class: Arachnida; order: Araneae; family: Linyphiidae; genus: Palliduphantes; taxonRank: species; taxonomicStatus: accepted; **Location:** higherGeography: North Africa; continent: Africa; country: Algeria; countryCode: DZ; municipality: Theniet El Had; verbatimElevation: 1474 m; verbatimCoordinateSystem: 35°51’58’’ N, 01°58’06’’E; **Identification:** identifiedBy: Ourida Kherbouche-Abrous; **Event:** samplingProtocol: pitfall traps; year: 2019; month: 2; day: 1-15
- **Type status:** Other material. **Occurrence:** recordedBy: Amina Saadi; individualCount: 3; sex: 2 males,1 female; lifeStage: adult; occurrenceID: DAA6EB10-E67B-5066-85DA-21F65EA532DB; **Taxon:** scientificNameID: urn:lsid:nmbe.ch:spidersp:011205; scientificName: *Palliduphanteslabilis* (Simon, 1913); kingdom: Animalia; phylum: Arthropoda; class: Arachnida; order: Araneae; family: Linyphiidae; genus: Palliduphantes; taxonRank: species; taxonomicStatus: accepted; **Location:** higherGeography: North Africa; continent: Africa; country: Algeria; countryCode: DZ; municipality: Theniet El Had; verbatimElevation: 1456 m; verbatimCoordinateSystem: 35°51’18’’ N, 01°59’20’’E; **Identification:** identifiedBy: Ourida Kherbouche-Abrous; **Event:** samplingProtocol: pitfall traps; year: 2019; month: 5; day: 1-15
- **Type status:** Other material. **Occurrence:** recordedBy: Amina Saadi; individualCount: 1; sex: 1 male; lifeStage: adult; occurrenceID: 46CCA381-0827-5C16-BD68-A80C65DBDE40; **Taxon:** scientificNameID: urn:lsid:nmbe.ch:spidersp:011205; scientificName: *Palliduphanteslabilis* (Simon, 1913); kingdom: Animalia; phylum: Arthropoda; class: Arachnida; order: Araneae; family: Linyphiidae; genus: Palliduphantes; taxonRank: species; taxonomicStatus: accepted; **Location:** higherGeography: North Africa; continent: Africa; country: Algeria; countryCode: DZ; municipality: Theniet El Had; verbatimElevation: 1449 m; verbatimCoordinateSystem: 35°51'34’’ N, 01°58’50’’E; **Identification:** identifiedBy: Ourida Kherbouche-Abrous; **Event:** samplingProtocol: pitfall traps; year: 2019; month: 6; day: 1-15
- **Type status:** Other material. **Occurrence:** recordedBy: Amina Saadi; individualCount: 2; sex: 1 male, 1 female; lifeStage: adult; occurrenceID: 48800744-BAFC-5C95-8069-4C22C3216FC4; **Taxon:** scientificNameID: urn:lsid:nmbe.ch:spidersp:011205; scientificName: *Palliduphanteslabilis* (Simon, 1913); kingdom: Animalia; phylum: Arthropoda; class: Arachnida; order: Araneae; family: Linyphiidae; genus: Palliduphantes; taxonRank: species; taxonomicStatus: accepted; **Location:** higherGeography: North Africa; continent: Africa; country: Algeria; countryCode: DZ; municipality: Theniet El Had; verbatimElevation: 1448 m; verbatimCoordinateSystem: 35°51'30’’ N, 01°59'11’’’E; **Identification:** identifiedBy: Ourida Kherbouche-Abrous; **Event:** samplingProtocol: pitfall traps; year: 2019; month: 6; day: 1-15
- **Type status:** Other material. **Occurrence:** recordedBy: Amina Saadi; individualCount: 4; sex: 2 males, 2 females; lifeStage: adult; occurrenceID: E322357E-8C40-5B24-ABF2-9403EF024E02; **Taxon:** scientificNameID: urn:lsid:nmbe.ch:spidersp:011205; scientificName: *Palliduphanteslabilis* (Simon, 1913); kingdom: Animalia; phylum: Arthropoda; class: Arachnida; order: Araneae; family: Linyphiidae; genus: Palliduphantes; taxonRank: species; taxonomicStatus: accepted; **Location:** higherGeography: North Africa; continent: Africa; country: Algeria; countryCode: DZ; municipality: Theniet El Had; verbatimElevation: 1448 m; verbatimCoordinateSystem: 35°51'30’’ N, 01°59'11’’’E; **Identification:** identifiedBy: Ourida Kherbouche-Abrous; **Event:** samplingProtocol: pitfall traps; year: 2019; month: 7; day: 1-15
- **Type status:** Other material. **Occurrence:** recordedBy: Amina Saadi; individualCount: 4; sex: 2 males, 2 females; lifeStage: adult; occurrenceID: F4BF7893-89F2-5985-A29B-B9245282C914; **Taxon:** scientificNameID: urn:lsid:nmbe.ch:spidersp:011205; scientificName: *Palliduphanteslabilis* (Simon, 1913); kingdom: Animalia; phylum: Arthropoda; class: Arachnida; order: Araneae; family: Linyphiidae; genus: Palliduphantes; taxonRank: species; taxonomicStatus: accepted; **Location:** higherGeography: North Africa; continent: Africa; country: Algeria; countryCode: DZ; municipality: Theniet El Had; verbatimElevation: 1449 m; verbatimCoordinateSystem: 35°51’34’’ N, 01°58’50’’ E; **Identification:** identifiedBy: Ourida Kherbouche-Abrous; **Event:** samplingProtocol: pitfall traps; year: 2019; month: 7; day: 1-15
- **Type status:** Other material. **Occurrence:** recordedBy: Amina Saadi; individualCount: 1; sex: 1 female; lifeStage: adult; occurrenceID: D1B08374-7917-5EDA-94F0-9E3A419C283E; **Taxon:** scientificNameID: urn:lsid:nmbe.ch:spidersp:011205; scientificName: *Palliduphanteslabilis* (Simon, 1913); kingdom: Animalia; phylum: Arthropoda; class: Arachnida; order: Araneae; family: Linyphiidae; genus: Palliduphantes; taxonRank: species; taxonomicStatus: accepted; **Location:** higherGeography: North Africa; continent: Africa; country: Algeria; countryCode: DZ; municipality: Theniet El Had; verbatimElevation: 1449 m; verbatimCoordinateSystem: 35°51’34’’ N, 01°58’50’’ E; **Identification:** identifiedBy: Ourida Kherbouche-Abrous; **Event:** samplingProtocol: pitfall traps; year: 2019; month: 10; day: 1-15
- **Type status:** Other material. **Occurrence:** recordedBy: Amina Saadi; individualCount: 3; sex: 1 males, 2 females; lifeStage: adult; occurrenceID: 4DBAB0F6-9025-5FA8-854F-A2F577CE996F; **Taxon:** scientificNameID: urn:lsid:nmbe.ch:spidersp:011205; scientificName: *Palliduphanteslabilis* (Simon, 1913); kingdom: Animalia; phylum: Arthropoda; class: Arachnida; order: Araneae; family: Linyphiidae; genus: Palliduphantes; taxonRank: species; taxonomicStatus: accepted; **Location:** higherGeography: North Africa; continent: Africa; country: Algeria; countryCode: DZ; municipality: Theniet El Had; verbatimElevation: 1456 m; verbatimCoordinateSystem: 35°51’18’’ N, 01°59’20’’E; **Identification:** identifiedBy: Ourida Kherbouche-Abrous; **Event:** samplingProtocol: pitfall traps; year: 2019; month: 10; day: 1-15

#### Distribution

**Algerian distribution**: The north part of the country (Simon 1913, Denis 1937, Bosmans 1985b, Deeleman-Reinhold 1985).

**Global distribution**: Algeria and Tunisia (World Spider Catalog 2024).

### Pelecopsis bucephala

(O. Pickard-Cambridge, 1875)

2C7E2563-6662-561C-8185-DBFA323A97A9

#### Materials

- **Type status:** Other material. **Occurrence:** recordedBy: Amina Saadi; individualCount: 2; sex: 2 males; lifeStage: adult; occurrenceID: 9543028F-C60C-55FF-B175-DE8892AABC2D; **Taxon:** scientificNameID: urn:lsid:nmbe.ch:spidersp:012248; scientificName: *Pelecopsisbucephala* (O. Pickard-Cambridge, 1875); kingdom: Animalia; phylum: Arthropoda; class: Arachnida; order: Araneae; family: Linyphiidae; genus: Pelecopsis; taxonRank: species; taxonomicStatus: accepted; **Location:** higherGeography: North Africa; continent: Africa; country: Algeria; countryCode: DZ; municipality: Theniet El Had; verbatimElevation: 1470 m; verbatimCoordinateSystem: 35°52’00’’ N, 01°58’07’’ E; **Identification:** identifiedBy: Ourida Kherbouche-Abrous; **Event:** samplingProtocol: pitfall traps; year: 2019; month: 5; day: 1-15
- **Type status:** Other material. **Occurrence:** recordedBy: Amina Saadi; individualCount: 1; sex: 1 male; lifeStage: adult; occurrenceID: E53C0ED7-A0FA-5F89-8C9B-4E00FD9D60F3; **Taxon:** scientificNameID: urn:lsid:nmbe.ch:spidersp:012248; scientificName: *Pelecopsisbucephala* (O. Pickard-Cambridge, 1875); kingdom: Animalia; phylum: Arthropoda; class: Arachnida; order: Araneae; family: Linyphiidae; genus: Pelecopsis; taxonRank: species; taxonomicStatus: accepted; **Location:** higherGeography: North Africa; continent: Africa; country: Algeria; countryCode: DZ; municipality: Theniet El Had; verbatimElevation: 1474 m; verbatimCoordinateSystem: 35°51’58’’ N, 01°58’06’’ E; **Identification:** identifiedBy: Ourida Kherbouche-Abrous; **Event:** samplingProtocol: pitfall traps; year: 2019; month: 6; day: 1-15
- **Type status:** Other material. **Occurrence:** recordedBy: Badis Bakhouche; individualCount: 1; sex: 1 female; lifeStage: adult; occurrenceID: CE6E8291-5CDD-5998-8209-F67430AC4C20; **Taxon:** scientificNameID: urn:lsid:nmbe.ch:spidersp:012248; scientificName: *Pelecopsisbucephala* (O. Pickard-Cambridge, 1875); kingdom: Animalia; phylum: Arthropoda; class: Arachnida; order: Araneae; family: Linyphiidae; genus: Pelecopsis; taxonRank: species; taxonomicStatus: accepted; **Location:** higherGeography: North Africa; continent: Africa; country: Algeria; countryCode: DZ; municipality: Theniet El Had; verbatimElevation: 1474 m; verbatimCoordinateSystem: 35°51’58’’ N, 01°58’06’’ E; **Identification:** identifiedBy: Ourida Kherbouche-Abrous; **Event:** samplingProtocol: hand collecting; year: 2019; month: 12; day: 16-31

#### Distribution

**Algerian distribution**: The north part from the east to the west (Denis 1968, Bosmans and Abrous 1992).

**Global distribution**: Western Mediterranean (World Spider Catalog 2024).

### Pelecopsis digitulus

Bosmans & Abrous, 1992

6C9E5C59-405B-5E71-B48B-D3576A676570

#### Materials

- **Type status:** Other material. **Occurrence:** recordedBy: Amina Saadi; individualCount: 2; sex: 2 males; lifeStage: adult; occurrenceID: 0A499E6B-3907-54F2-8006-1E98906FF1B3; **Taxon:** scientificNameID: urn:lsid:nmbe.ch:spidersp:012254; scientificName: *Pelecopsisdigitulus* Bosmans & Abrous, 1992; kingdom: Animalia; phylum: Arthropoda; class: Arachnida; order: Araneae; family: Linyphiidae; genus: Pelecopsis ; taxonRank: species; taxonomicStatus: accepted; **Location:** higherGeography: North Africa; continent: Africa; country: Algeria; countryCode: DZ; municipality: Theniet El Had; verbatimElevation: 1448 m; verbatimCoordinateSystem: 35°51’30’’ N, 01°59’11’’ E; **Identification:** identifiedBy: Ourida Kherbouche-Abrous; **Event:** samplingProtocol: pitfall traps; year: 2019; month: 2; day: 1-15
- **Type status:** Other material. **Occurrence:** recordedBy: Amina Saadi; individualCount: 7; sex: 4 males, 3 females; lifeStage: adult; occurrenceID: DA55792A-6836-55AF-B02B-14379FD29839; **Taxon:** scientificNameID: urn:lsid:nmbe.ch:spidersp:012254; scientificName: *Pelecopsisdigitulus* Bosmans & Abrous, 1992; kingdom: Animalia; phylum: Arthropoda; class: Arachnida; order: Araneae; family: Linyphiidae; genus: Pelecopsis ; taxonRank: species; taxonomicStatus: accepted; **Location:** higherGeography: North Africa; continent: Africa; country: Algeria; countryCode: DZ; municipality: Theniet El Had; verbatimElevation: 1456 m; verbatimCoordinateSystem: 35°51’18’’ N, 01°59’20’’ E; **Identification:** identifiedBy: Ourida Kherbouche-Abrous; **Event:** samplingProtocol: pitfall traps; year: 2019; month: 4; day: 1-15

#### Distribution

**Algerian distribution**: Interior of the country (Bosmans and Abrous 1992).

**Global distribution**: Algeria and France (Corsica), Italy (World Spider Catalog 2024).

### Pelecopsis inedita

(O. Pickard-Cambridge, 1875)

B1AF7C50-5120-53B5-9DC2-9D9A732F8FE0

#### Materials

- **Type status:** Other material. **Occurrence:** recordedBy: Amina Saadi; individualCount: 2; sex: 2 males; lifeStage: adult; occurrenceID: 82E9AA40-0253-5EAD-908D-250121F6856C; **Taxon:** scientificNameID: urn:lsid:nmbe.ch:spidersp:012264; scientificName: *Pelecopsisinedita* (O. Pickard-Cambridge, 1875); kingdom: Animalia ; phylum: Arthropoda ; class: Arachnida ; order: Araneae; family: Linyphiidae; genus: Pelecopsis; taxonRank: species; taxonomicStatus: accepted; **Location:** higherGeography: North Africa; continent: Africa; country: Algeria; countryCode: DZ; municipality: Theniet El Had; verbatimElevation: 1448 m; verbatimCoordinateSystem: 35°51'30'' N, 01°59'11'' E; **Identification:** identifiedBy: Ourida Kherbouche-Abrous; **Event:** samplingProtocol: pitfall traps; year: 2019; month: 1; day: 1-15
- **Type status:** Other material. **Occurrence:** recordedBy: Amina Saadi; individualCount: 1; sex: 1 female; lifeStage: adult; occurrenceID: A286C028-BF53-53D5-B36D-148F4046E945; **Taxon:** scientificNameID: urn:lsid:nmbe.ch:spidersp:012264; scientificName: *Pelecopsisinedita* (O. Pickard-Cambridge, 1875); kingdom: Animalia ; phylum: Arthropoda ; class: Arachnida ; order: Araneae; family: Linyphiidae; genus: Pelecopsis; taxonRank: species; taxonomicStatus: accepted; **Location:** higherGeography: North Africa; continent: Africa; country: Algeria; countryCode: DZ; municipality: Theniet El Had; verbatimElevation: 1448 m; verbatimCoordinateSystem: 35°51'30'' N, 01°59'11'' E; **Identification:** identifiedBy: Ourida Kherbouche-Abrous; **Event:** samplingProtocol: pitfall traps; year: 2019; month: 4; day: 1-15
- **Type status:** Other material. **Occurrence:** recordedBy: Badis Bakhouche; individualCount: 2; sex: 1 male, 1 female; lifeStage: adult; occurrenceID: 20003C8D-74C9-5E3F-92E3-B305458F86D2; **Taxon:** scientificNameID: urn:lsid:nmbe.ch:spidersp:012264; scientificName: *Pelecopsisinedita* (O. Pickard-Cambridge, 1875); kingdom: Animalia ; phylum: Arthropoda ; class: Arachnida ; order: Araneae; family: Linyphiidae; genus: Pelecopsis; taxonRank: species; taxonomicStatus: accepted; **Location:** higherGeography: North Africa; continent: Africa; country: Algeria; countryCode: DZ; municipality: Theniet El Had; verbatimElevation: 1470 m; verbatimCoordinateSystem: 35°52'00'' N, 01°58'07'' E; **Identification:** identifiedBy: Ourida Kherbouche-Abrous; **Event:** samplingProtocol: hand collecting; year: 2019; month: 7; day: 1-15
- **Type status:** Other material. **Occurrence:** recordedBy: Amina Saadi; individualCount: 1; sex: 1 male; lifeStage: adult; occurrenceID: A0FE9014-4230-5D14-A45F-E5BCDAC33E0B; **Taxon:** scientificNameID: urn:lsid:nmbe.ch:spidersp:012264; scientificName: *Pelecopsisinedita* (O. Pickard-Cambridge, 1875); kingdom: Animalia ; phylum: Arthropoda ; class: Arachnida ; order: Araneae; family: Linyphiidae; genus: Pelecopsis; taxonRank: species; taxonomicStatus: accepted; **Location:** higherGeography: North Africa; continent: Africa; country: Algeria; countryCode: DZ; municipality: Theniet El Had; verbatimElevation: 1456 m; verbatimCoordinateSystem: 35°51'18'' N, 01°59'20'' E; **Identification:** identifiedBy: Ourida Kherbouche-Abrous; **Event:** samplingProtocol: pitfall traps; year: 2019; month: 10; day: 1-15
- **Type status:** Other material. **Occurrence:** recordedBy: Amina Saadi; individualCount: 1; sex: 1 male; lifeStage: adult; occurrenceID: 9A69DCF8-D783-5528-88A9-2826FC0B4E26; **Taxon:** scientificNameID: urn:lsid:nmbe.ch:spidersp:012264; scientificName: *Pelecopsisinedita* (O. Pickard-Cambridge, 1875); kingdom: Animalia ; phylum: Arthropoda ; class: Arachnida ; order: Araneae; family: Linyphiidae; genus: Pelecopsis; taxonRank: species; taxonomicStatus: accepted; **Location:** higherGeography: North Africa; continent: Africa; country: Algeria; countryCode: DZ; municipality: Theniet El Had; verbatimElevation: 1448 m; verbatimCoordinateSystem: 35°51'30'' N, 01°59'11'' E; **Identification:** identifiedBy: Ourida Kherbouche-Abrous; **Event:** samplingProtocol: pitfall traps; year: 2019; month: 12; day: 16-31
- **Type status:** Other material. **Occurrence:** recordedBy: Amina Saadi; individualCount: 1; sex: 1 female; lifeStage: adult; occurrenceID: 40BF0BA0-623D-57FD-A824-CFD6964428D7; **Taxon:** scientificNameID: urn:lsid:nmbe.ch:spidersp:012264; scientificName: *Pelecopsisinedita* (O. Pickard-Cambridge, 1875); kingdom: Animalia ; phylum: Arthropoda ; class: Arachnida ; order: Araneae; family: Linyphiidae; genus: Pelecopsis; taxonRank: species; taxonomicStatus: accepted; **Location:** higherGeography: North Africa; continent: Africa; country: Algeria; countryCode: DZ; municipality: Theniet El Had; verbatimElevation: 1474 m; verbatimCoordinateSystem: 35°51'58'' N, 01°58'06'' E; **Identification:** identifiedBy: Ourida Kherbouche-Abrous; **Event:** samplingProtocol: pitfall traps; year: 2019; month: 12; day: 16-31

#### Distribution

**Algerian distribution**: North, east and west of the country (Denis 1962, Bosmans and Abrous 1992, Bouseksou et al. 2015).

**Global distribution**: Canary Islands, Mediterranean (World Spider Catalog 2024).

### Pelecopsis major

(Denis, 1945)

D66940A2-18F9-52D7-A03C-95697B017635

#### Materials

- **Type status:** Other material. **Occurrence:** recordedBy: Badis Bakhouche; individualCount: 1; sex: 1 female; lifeStage: adult; occurrenceID: 01AAD7AA-3ADA-5B46-9CE9-F071C9467FCF; **Taxon:** scientificNameID: urn:lsid:nmbe.ch:spidersp:012275; scientificName: *Pelecopsismajor* (Denis, 1945); kingdom: Animalia; phylum: Arthropoda; class: Arachnida; order: Araneae; family: Linyphiidae; genus: Pelecopsis; taxonRank: species; taxonomicStatus: accepted; **Location:** higherGeography: North Africa; continent: Africa; country: Algeria; countryCode: DZ; municipality: Theniet El Had; verbatimElevation: 1470 m; verbatimCoordinateSystem: 35°52’00’’ N, 01°58’07’’ E; **Identification:** identifiedBy: Ourida Kherbouche-Abrous; **Event:** samplingProtocol: hand collecting; year: 2019; month: 1; day: 1-15
- **Type status:** Other material. **Occurrence:** recordedBy: Amina Saadi; individualCount: 2; sex: 2 females; lifeStage: adult; occurrenceID: 2F74A063-3C56-5ECD-85DB-7965F6007FCA; **Taxon:** scientificNameID: urn:lsid:nmbe.ch:spidersp:012275; scientificName: *Pelecopsismajor* (Denis, 1945); kingdom: Animalia; phylum: Arthropoda; class: Arachnida; order: Araneae; family: Linyphiidae; genus: Pelecopsis; taxonRank: species; taxonomicStatus: accepted; **Location:** higherGeography: North Africa; continent: Africa; country: Algeria; countryCode: DZ; municipality: Theniet El Had; verbatimElevation: 1448 m; verbatimCoordinateSystem: 35°51’30’’ N, 01°59’11’’ E; **Identification:** identifiedBy: Ourida Kherbouche-Abrous; **Event:** samplingProtocol: pitfall traps; year: 2019; month: 1; day: 1-15
- **Type status:** Other material. **Occurrence:** recordedBy: Amina Saadi; individualCount: 2; sex: 1 male, 1 female; lifeStage: adult; occurrenceID: 2BDDC5AF-13FD-51B2-BB85-71E3A48E7B1F; **Taxon:** scientificNameID: urn:lsid:nmbe.ch:spidersp:012275; scientificName: *Pelecopsismajor* (Denis,1945); kingdom: Animalia; phylum: Arthropoda; class: Arachnida; order: Araneae; family: Linyphiidae; genus: Pelecopsis; taxonRank: species; taxonomicStatus: accepted; **Location:** higherGeography: North Africa; continent: Africa; country: Algeria; countryCode: DZ; municipality: Theniet El Had; verbatimElevation: 1449 m; verbatimCoordinateSystem: 35°51’34’’ N, 01°58’50’’ E; **Identification:** identifiedBy: Ourida Kherbouche-Abrous; **Event:** samplingProtocol: pitfall traps; year: 2019; month: 1; day: 1-15
- **Type status:** Other material. **Occurrence:** recordedBy: Badis Bakhouche; individualCount: 2; sex: 2 females; lifeStage: adult; occurrenceID: 22552454-F0A8-552F-A8CD-2DF81E2DF824; **Taxon:** scientificNameID: urn:lsid:nmbe.ch:spidersp:012275; scientificName: *Pelecopsismajor* (Denis, 1945); kingdom: Animalia; phylum: Arthropoda; class: Arachnida; order: Araneae; family: Linyphiidae; genus: Pelecopsis; taxonRank: species; taxonomicStatus: accepted; **Location:** higherGeography: North Africa; continent: Africa; country: Algeria; countryCode: DZ; municipality: Theniet El Had; verbatimElevation: 1448 m; verbatimCoordinateSystem: 35°51’30’’ N, 01°59’11’’ E; **Identification:** identifiedBy: Ourida Kherbouche-Abrous; **Event:** samplingProtocol: pitfall traps; year: 2019; month: 2; day: 1-15
- **Type status:** Other material. **Occurrence:** recordedBy: Amina Saadi; individualCount: 2; sex: 1 male, 1 female; lifeStage: adult; occurrenceID: 791AC47A-2E28-5962-B9F2-A397F76B0589; **Taxon:** scientificNameID: urn:lsid:nmbe.ch:spidersp:012275; scientificName: *Pelecopsismajor* (Denis, 1945); kingdom: Animalia; phylum: Arthropoda; class: Arachnida; order: Araneae; family: Linyphiidae; genus: Pelecopsis; taxonRank: species; taxonomicStatus: accepted; **Location:** higherGeography: North Africa; continent: Africa; country: Algeria; countryCode: DZ; municipality: Theniet El Had; verbatimElevation: 1448 m; verbatimCoordinateSystem: 35°51’30’’ N, 01°59’11’’ E; **Identification:** identifiedBy: Ourida Kherbouche-Abrous; **Event:** samplingProtocol: pitfall traps; year: 2019; month: 2; day: 1-15
- **Type status:** Other material. **Occurrence:** recordedBy: Badis Bakhouche; individualCount: 1; sex: 1 male; lifeStage: adult; occurrenceID: DEDDA596-B270-589F-98AA-FE1D73B5B052; **Taxon:** scientificNameID: urn:lsid:nmbe.ch:spidersp:012275; scientificName: *Pelecopsismajor* (Denis, 1945); kingdom: Animalia; phylum: Arthropoda; class: Arachnida; order: Araneae; family: Linyphiidae; genus: Pelecopsis; taxonRank: species; taxonomicStatus: accepted; **Location:** higherGeography: North Africa; continent: Africa; country: Algeria; countryCode: DZ; municipality: Theniet El Had; verbatimElevation: 1448 m; verbatimCoordinateSystem: 35°51’30’’ N, 01°59’11’’ E; **Identification:** identifiedBy: Ourida Kherbouche-Abrous; **Event:** samplingProtocol: hand collecting; year: 2019; month: 4; day: 1-15
- **Type status:** Other material. **Occurrence:** recordedBy: Amina Saadi; individualCount: 1; sex: 1 male; lifeStage: adult; occurrenceID: 5DA69261-F940-5369-8672-A25090346185; **Taxon:** scientificNameID: urn:lsid:nmbe.ch:spidersp:012275; scientificName: *Pelecopsismajor* (Denis, 1945); kingdom: Animalia; phylum: Arthropoda; class: Arachnida; order: Araneae; family: Linyphiidae; genus: Pelecopsis; taxonRank: species; taxonomicStatus: accepted; **Location:** higherGeography: North Africa; continent: Africa; country: Algeria; countryCode: DZ; municipality: Theniet El Had; verbatimElevation: 1456 m; verbatimCoordinateSystem: 35°51’18’’ N, 01°59’20’’ E; **Identification:** identifiedBy: Ourida Kherbouche-Abrous; **Event:** samplingProtocol: pitfall traps; year: 2019; month: 10; day: 1-15
- **Type status:** Other material. **Occurrence:** recordedBy: Amina Saadi; individualCount: 1; sex: 1 male; lifeStage: adult; occurrenceID: A3FECF1E-1C11-58E6-9E99-FF00A579E763; **Taxon:** scientificNameID: urn:lsid:nmbe.ch:spidersp:012275; scientificName: *Pelecopsismajor* (Denis, 1945); kingdom: Animalia; phylum: Arthropoda; class: Arachnida; order: Araneae; family: Linyphiidae; genus: Pelecopsis; taxonRank: species; taxonomicStatus: accepted; **Location:** higherGeography: North Africa; continent: Africa; country: Algeria; countryCode: DZ; municipality: Theniet El Had; verbatimElevation: 1449 m; verbatimCoordinateSystem: 35°51’34’’ N, 01°58’50’’ E; **Identification:** identifiedBy: Ourida Kherbouche-Abrous; **Event:** samplingProtocol: pitfall traps; year: 2019; month: 10; day: 1-15
- **Type status:** Other material. **Occurrence:** recordedBy: Amina Saadi; individualCount: 7; sex: 5 males, 2 females; lifeStage: adult; occurrenceID: 72BC7982-9841-509E-A066-29CE2A148D87; **Taxon:** scientificNameID: urn:lsid:nmbe.ch:spidersp:012275; scientificName: *Pelecopsismajor* (Denis, 1945); kingdom: Animalia; phylum: Arthropoda; class: Arachnida; order: Araneae; family: Linyphiidae; genus: Pelecopsis; taxonRank: species; taxonomicStatus: accepted; **Location:** higherGeography: North Africa; continent: Africa; country: Algeria; countryCode: DZ; municipality: Theniet El Had; verbatimElevation: 1470 m; verbatimCoordinateSystem: 35°52’00’’ N, 01°58’07’’ E; **Identification:** identifiedBy: Ourida Kherbouche-Abrous; **Event:** samplingProtocol: pitfall traps; year: 2019; month: 10; day: 1-15
- **Type status:** Other material. **Occurrence:** recordedBy: Amina Saadi; individualCount: 3; sex: 3 females; lifeStage: adult; occurrenceID: 45B1FB71-F16C-5BE7-A52D-B1FBCC3D4BE2; **Taxon:** scientificNameID: urn:lsid:nmbe.ch:spidersp:012275; scientificName: *Pelecopsismajor* (Denis, 1945); kingdom: Animalia; phylum: Arthropoda; class: Arachnida; order: Araneae; family: Linyphiidae; genus: Pelecopsis; taxonRank: species; taxonomicStatus: accepted; **Location:** higherGeography: North Africa; continent: Africa; country: Algeria; countryCode: DZ; municipality: Theniet El Had; verbatimElevation: 1470 m; verbatimCoordinateSystem: 35°52’00’’ N, 01°58’07’’ E; **Identification:** identifiedBy: Ourida Kherbouche-Abrous; **Event:** samplingProtocol: pitfall traps; year: 2019; month: 11; day: 1-15
- **Type status:** Other material. **Occurrence:** recordedBy: Amina Saadi; individualCount: 8; sex: 8 males; lifeStage: adult; occurrenceID: 9AE68F45-F568-5740-96F3-554051E6889B; **Taxon:** scientificNameID: urn:lsid:nmbe.ch:spidersp:012275; scientificName: *Pelecopsismajor* (Denis, 1945); kingdom: Animalia; phylum: Arthropoda; class: Arachnida; order: Araneae; family: Linyphiidae; genus: Pelecopsis; taxonRank: species; taxonomicStatus: accepted; **Location:** higherGeography: North Africa; continent: Africa; country: Algeria; countryCode: DZ; municipality: Theniet El Had; verbatimElevation: 1470 m; verbatimCoordinateSystem: 35°52’00’’ N, 01°58’07’’ E; **Identification:** identifiedBy: Ourida Kherbouche-Abrous; **Event:** samplingProtocol: pitfall traps; year: 2019; month: 12; day: 1-15
- **Type status:** Other material. **Occurrence:** recordedBy: Amina Saadi; individualCount: 8; sex: 8 males; lifeStage: adult; occurrenceID: A9342886-0E42-5622-9CA2-308683EC169B; **Taxon:** scientificNameID: urn:lsid:nmbe.ch:spidersp:012275; scientificName: *elecopsis major* (Denis, 1945); kingdom: Animalia; phylum: Arthropoda; class: Arachnida; order: Araneae; family: Linyphiidae; genus: Pelecopsis; taxonRank: species; taxonomicStatus: accepted; **Location:** higherGeography: North Africa; continent: Africa; country: Algeria; countryCode: DZ; municipality: Theniet El Had; verbatimElevation: 1449 m; verbatimCoordinateSystem: 35°51’34’’ N, 01°58’50’’ E; **Identification:** identifiedBy: Ourida Kherbouche-Abrous; **Event:** samplingProtocol: pitfall traps; year: 2019; month: 12; day: 1-15

#### Distribution

**Algerian distribution**: North part of the country (Denis 1945a, Bosmans and Abrous 1992).

**Global distribution**: Algeria (World Spider Catalog 2024).

### Pelecopsis oranensis

(Simon, 1884)

31B6B3E3-B70C-521E-BC52-12367E419743

#### Materials

- **Type status:** Other material. **Occurrence:** recordedBy: Amina Saadi; individualCount: 1; sex: 1 male; lifeStage: adult; occurrenceID: 3793DBE9-B349-5CE8-9817-9C324A8B0A5C; **Taxon:** scientificNameID: urn:lsid:nmbe.ch:spidersp:012288; scientificName: *Pelecopsisoranensis* (Simon, 1884); kingdom: Animalia; phylum: Arthropoda; class: Arachnida; order: Araneae; family: Linyphiidae; genus: Pelecopsis; taxonRank: species; taxonomicStatus: accepted; **Location:** higherGeography: North Africa; continent: Africa; country: Algeria; countryCode: DZ; municipality: Theniet El Had; verbatimElevation: 1448 m; verbatimCoordinateSystem: 35°51’30’’ N, 01°59’11’’ E; **Identification:** identifiedBy: Ourida Kherbouche-Abrous; **Event:** samplingProtocol: pitfall traps; year: 2019; month: 2; day: 1-15
- **Type status:** Other material. **Occurrence:** recordedBy: Amina Saadi; individualCount: 1; sex: 1 male; lifeStage: adult; occurrenceID: BC143B64-6348-5784-A81F-5CF866730EC7; **Taxon:** scientificNameID: urn:lsid:nmbe.ch:spidersp:012288; scientificName: *Pelecopsisoranensis* (Simon, 1884); kingdom: Animalia; phylum: Arthropoda; class: Arachnida; order: Araneae; family: Linyphiidae; genus: Pelecopsis; taxonRank: species; taxonomicStatus: accepted; **Location:** higherGeography: North Africa; continent: Africa; country: Algeria; countryCode: DZ; municipality: Theniet El Had; verbatimElevation: 1474 m; verbatimCoordinateSystem: 35°51’58’’ N, 01°58’06’’E; **Identification:** identifiedBy: Ourida Kherbouche-Abrous; **Event:** samplingProtocol: pitfall traps; year: 2019; month: 12; day: 16-31

#### Distribution

**Algerian distribution**: The north part of the country (Simon 1884, Denis 1962, Bosmans and Abrous 1992).

**Global distribution**: Morocco and Algeria (World Spider Catalog 2024).

### Pelecopsis oujda

Bosmans & Abrous, 1992

B7390ADB-F097-5269-AD20-5C04E3BCA4C4

#### Materials

- **Type status:** Other material. **Occurrence:** recordedBy: Amina Saadi; individualCount: 1; sex: 1 male; lifeStage: adult; occurrenceID: 8911B7C8-E806-558D-A0F6-AFF051FB93B6; **Taxon:** scientificNameID: urn:lsid:nmbe.ch:spidersp:012289; scientificName: *Pelecopsisoujda* Bosmans & Abrous, 1992; kingdom: Animalia ; phylum: Arthropoda ; class: Arachnida ; order: Araneae ; family: Linyphiidae ; genus: Pelecopsis ; taxonRank: species; taxonomicStatus: accepted; **Location:** higherGeography: North Africa; continent: Africa; country: Algeria; countryCode: DZ; municipality: Theniet El Had; verbatimElevation: 1470 m; verbatimCoordinateSystem: 35°52’00’’ N, 01°58’07’’ E; **Identification:** identifiedBy: Ourida Kherbouche-Abrous; Robert Bosmans; **Event:** samplingProtocol: pitfall traps; year: 2019; month: 1; day: 1-15

#### Distribution

**Algerian distribution**: To date, the species is only known from Théniet El Had National Park.

**Global distribution**: Morocco (World Spider Catalog 2024) and Algeria (First record).

### Sintula penicilliger

(Simon, 1884)

BA36621D-5503-5980-AF3E-ACA9036C57D1

#### Materials

- **Type status:** Other material. **Occurrence:** recordedBy: Amina Saadi; individualCount: 2; sex: 2 males; lifeStage: adult; occurrenceID: EEC5473A-189C-5E88-92B3-3D54FE1944C5; **Taxon:** scientificNameID: urn:lsid:nmbe.ch:spidersp:012650; scientificName: *Sintulapenicilliger* (Simon, 1884); kingdom: Animalia; phylum: Arthropoda; class: Arachnida; order: Araneae; family: Linyphiidae; genus: Sintula; taxonRank: species; taxonomicStatus: accepted; **Location:** higherGeography: North Africa; continent: Africa; country: Algeria; countryCode: DZ; municipality: Theniet El Had; verbatimElevation: 1456 m; verbatimCoordinateSystem: 35°51’18’’ N, 01°59’20’’ E; **Identification:** identifiedBy: Ourida Kherbouche-Abrous; **Event:** samplingProtocol: pitfall traps; year: 2019; month: 1; day: 1-15
- **Type status:** Other material. **Occurrence:** recordedBy: Amina Saadi; individualCount: 2; sex: 2 males; lifeStage: adult; occurrenceID: 7A45CD37-8A8F-5EF3-9E72-7595DAFAB0DE; **Taxon:** scientificNameID: urn:lsid:nmbe.ch:spidersp:012650; scientificName: *Sintulapenicilliger* (Simon, 1884); kingdom: Animalia; phylum: Arthropoda; class: Arachnida; order: Araneae; family: Linyphiidae; genus: Sintula; taxonRank: species; taxonomicStatus: accepted; **Location:** higherGeography: North Africa; continent: Africa; country: Algeria; countryCode: DZ; municipality: Theniet El Had; verbatimElevation: 1470 m; verbatimCoordinateSystem: 35°52’00’’ N, 01°58’07’’ E; **Identification:** identifiedBy: Ourida Kherbouche-Abrous; **Event:** samplingProtocol: pitfall traps; year: 2019; month: 2; day: 1-15
- **Type status:** Other material. **Occurrence:** recordedBy: Badis Bakhouche; individualCount: 1; sex: 1 male; lifeStage: adult; occurrenceID: 7E903115-FB99-5C30-9CAC-EA32326A311A; **Taxon:** scientificNameID: urn:lsid:nmbe.ch:spidersp:012650; scientificName: *Sintulapenicilliger* (Simon, 1884); kingdom: Animalia; phylum: Arthropoda; class: Arachnida; order: Araneae; family: Linyphiidae; genus: Sintula; taxonRank: species; taxonomicStatus: accepted; **Location:** higherGeography: North Africa; continent: Africa; country: Algeria; countryCode: DZ; municipality: Theniet El Had; verbatimElevation: 1456 m; verbatimCoordinateSystem: 35°51’18’’ N, 01°59’20’’ E; **Identification:** identifiedBy: Ourida Kherbouche-Abrous; **Event:** samplingProtocol: hand collecting; year: 2019; month: 4; day: 1-15
- **Type status:** Other material. **Occurrence:** recordedBy: Amina Saadi; individualCount: 4; sex: 4 males; lifeStage: adult; occurrenceID: C56FBAD9-327B-5CB3-AF00-1FF1F32C4A7A; **Taxon:** scientificNameID: urn:lsid:nmbe.ch:spidersp:012650; scientificName: *Sintulapenicilliger* (Simon, 1884); kingdom: Animalia; phylum: Arthropoda; class: Arachnida; order: Araneae; family: Linyphiidae; genus: Sintula; taxonRank: species; taxonomicStatus: accepted; **Location:** higherGeography: North Africa; continent: Africa; country: Algeria; countryCode: DZ; municipality: Theniet El Had; verbatimElevation: 1474 m; verbatimCoordinateSystem: 35°51’58’’ N, 01°58’06’’E; **Identification:** identifiedBy: Ourida Kherbouche-Abrous; **Event:** samplingProtocol: hand collecting; year: 2019; month: 7; day: 1-15
- **Type status:** Other material. **Occurrence:** recordedBy: Amina Saadi; individualCount: 4; sex: 2 males, 2 females; lifeStage: adult; occurrenceID: 561DDB39-4F01-58F3-AF78-EFC32F0091C5; **Taxon:** scientificNameID: urn:lsid:nmbe.ch:spidersp:012650; scientificName: *Sintulapenicilliger* (Simon, 1884); kingdom: Animalia; phylum: Arthropoda; class: Arachnida; order: Araneae; family: Linyphiidae; genus: Sintula; taxonRank: species; taxonomicStatus: accepted; **Location:** higherGeography: North Africa; continent: Africa; country: Algeria; countryCode: DZ; municipality: Theniet El Had; verbatimElevation: 1470 m; verbatimCoordinateSystem: 35°52’00’’ N, 01°58’07’’ E; **Identification:** identifiedBy: Ourida Kherbouche-Abrous; **Event:** samplingProtocol: pitfall traps; year: 2019; month: 12; day: 1-15

#### Distribution

**Algerian distribution**: North of the country (Bosmans 1991a).

**Global Distribution**: Algeria (World Spider Catalog 2024).

### Tenuiphantes tenuis

(Blackwall, 1852)

4D45C85B-EF10-548C-B3FD-DB98886D0971

#### Materials

- **Type status:** Other material. **Occurrence:** recordedBy: Badis Bakhouche; individualCount: 1; sex: female; lifeStage: adult; occurrenceID: 96E58A33-49F1-5640-B20B-1894F5BBBDB2; **Taxon:** scientificNameID: urn:lsid:nmbe.ch:spidersp:012897; scientificName: *Tenuiphantestenuis* (Blackwall, 1852); kingdom: Animalia; phylum: Arthropoda; class: Arachnida; order: Araneae; family: Linyphiidae; genus: Tenuiphantes; taxonRank: species; taxonomicStatus: accepted; **Location:** higherGeography: North Africa; continent: Africa; country: Algeria; countryCode: DZ; municipality: Theniet El Had; verbatimElevation: 1474 m; verbatimCoordinateSystem: 35°51’58’’ N, 01°58’06’’E; **Identification:** identifiedBy: Ourida Kherbouche-Abrous; **Event:** samplingProtocol: hand collecting; year: 2019; month: 3; day: 1-15
- **Type status:** Other material. **Occurrence:** recordedBy: Amina Saadi; individualCount: 2; sex: 1 male, 1 female; lifeStage: adult; occurrenceID: E816F9B9-68FA-5024-8FAD-39F71659ECC7; **Taxon:** scientificNameID: urn:lsid:nmbe.ch:spidersp:012897; scientificName: *Tenuiphantestenuis* (Blackwall, 1852); kingdom: Animalia; phylum: Arthropoda; class: Arachnida; order: Araneae; family: Linyphiidae; genus: Tenuiphantes; taxonRank: species; taxonomicStatus: accepted; **Location:** higherGeography: North Africa; continent: Africa; country: Algeria; countryCode: DZ; municipality: Theniet El Had; verbatimElevation: 1448 m; verbatimCoordinateSystem: 35°51’30’’ N, 01°59’11’’ E; **Identification:** identifiedBy: Ourida Kherbouche-Abrous; **Event:** samplingProtocol: pitfall traps; year: 2019; month: 4; day: 1-15
- **Type status:** Other material. **Occurrence:** recordedBy: Amina Saadi; individualCount: 1; sex: 1 female; lifeStage: adult; occurrenceID: E816F9B9-68FA-5024-8FAD-39F71659ECC7; **Taxon:** scientificNameID: urn:lsid:nmbe.ch:spidersp:012897; scientificName: *Tenuiphantestenuis* (Blackwall, 1852); kingdom: Animalia; phylum: Arthropoda; class: Arachnida; order: Araneae; family: Linyphiidae; genus: Tenuiphantes; taxonRank: species; taxonomicStatus: accepted; **Location:** higherGeography: North Africa; continent: Africa; country: Algeria; countryCode: DZ; municipality: Theniet El Had; verbatimElevation: 1448 m; verbatimCoordinateSystem: 35°51’30’’ N, 01°59’11’’ E; **Identification:** identifiedBy: Ourida Kherbouche-Abrous; **Event:** samplingProtocol: pitfall traps; year: 2019; month: 5; day: 1-15
- **Type status:** Other material. **Occurrence:** recordedBy: Amina Saadi; individualCount: 1; sex: 1 male; lifeStage: adult; occurrenceID: 6E73AD13-BA28-592F-B0B9-EDDD19087711; **Taxon:** scientificNameID: urn:lsid:nmbe.ch:spidersp:012897; scientificName: *Tenuiphantestenuis* (Blackwall, 1852); kingdom: Animalia; phylum: Arthropoda; class: Arachnida; order: Araneae; family: Linyphiidae; genus: Tenuiphantes; taxonRank: species; taxonomicStatus: accepted; **Location:** higherGeography: North Africa; continent: Africa; country: Algeria; countryCode: DZ; municipality: Theniet El Had; verbatimElevation: 1448 m; verbatimCoordinateSystem: 35°51’30’’ N, 01°59’11’’ E; **Identification:** identifiedBy: Ourida Kherbouche-Abrous; **Event:** samplingProtocol: pitfall traps; year: 2019; month: 6; day: 1-15
- **Type status:** Other material. **Occurrence:** recordedBy: Amina Saadi; individualCount: 3; sex: 1 male, 2 females; lifeStage: adult; occurrenceID: 8D65FDA0-7703-526C-896E-020B3D3A29CA; **Taxon:** scientificNameID: urn:lsid:nmbe.ch:spidersp:012897; scientificName: *Tenuiphantestenuis* (Blackwall, 1852); kingdom: Animalia; phylum: Arthropoda; class: Arachnida; order: Araneae; family: Linyphiidae; genus: Tenuiphantes; taxonRank: species; taxonomicStatus: accepted; **Location:** higherGeography: North Africa; continent: Africa; country: Algeria; countryCode: DZ; municipality: Theniet El Had; verbatimElevation: 1448 m; verbatimCoordinateSystem: 35°51’30’’ N, 01°59’11’’ E; **Identification:** identifiedBy: Ourida Kherbouche-Abrous; **Event:** samplingProtocol: pitfall traps; year: 2019; month: 7; day: 1-15
- **Type status:** Other material. **Occurrence:** recordedBy: Amina Saadi; individualCount: 12; sex: 8 males, 4 females; lifeStage: adult; occurrenceID: BB684436-1AB7-5446-8C65-C6129C47A539; **Taxon:** scientificNameID: urn:lsid:nmbe.ch:spidersp:012897; scientificName: *Tenuiphantestenuis* (Blackwall, 1852); kingdom: Animalia; phylum: Arthropoda; class: Arachnida; order: Araneae; family: Linyphiidae; genus: Tenuiphantes; taxonRank: species; taxonomicStatus: accepted; **Location:** higherGeography: North Africa; continent: Africa; country: Algeria; countryCode: DZ; municipality: Theniet El Had; verbatimElevation: 1474 m; verbatimCoordinateSystem: 35°51’58’’ N, 01°58’06’’E; **Identification:** identifiedBy: Ourida Kherbouche-Abrous; **Event:** samplingProtocol: pitfall traps; year: 2019; month: 7; day: 1-15
- **Type status:** Other material. **Occurrence:** recordedBy: Amina Saadi; individualCount: 1; sex: 1 male; lifeStage: adult; occurrenceID: 9C782075-6D6C-5FFC-87FC-7E7043B6DAED; **Taxon:** scientificNameID: urn:lsid:nmbe.ch:spidersp:012897; scientificName: *Tenuiphantestenuis* (Blackwall, 1852); kingdom: Animalia; phylum: Arthropoda; class: Arachnida; order: Araneae; family: Linyphiidae; genus: Tenuiphantes; taxonRank: species; taxonomicStatus: accepted; **Location:** higherGeography: North Africa; continent: Africa; country: Algeria; countryCode: DZ; municipality: Theniet El Had; verbatimElevation: 1448 m; verbatimCoordinateSystem: 35°51’30’’ N, 01°59’11’’ E; **Identification:** identifiedBy: Ourida Kherbouche-Abrous; **Event:** samplingProtocol: pitfall traps; year: 2019; month: 8; day: 1-15
- **Type status:** Other material. **Occurrence:** recordedBy: Amina Saadi; individualCount: 16; sex: 12 males, 4 males; lifeStage: adult; occurrenceID: 921A2219-FD5A-5782-A1D2-58969D5303F8; **Taxon:** scientificNameID: urn:lsid:nmbe.ch:spidersp:012897; scientificName: *Tenuiphantestenuis* (Blackwall, 1852); kingdom: Animalia; phylum: Arthropoda; class: Arachnida; order: Araneae; family: Linyphiidae; genus: Tenuiphantes; taxonRank: species; taxonomicStatus: accepted; **Location:** higherGeography: North Africa; continent: Africa; country: Algeria; countryCode: DZ; municipality: Theniet El Had; verbatimElevation: 1470 m; verbatimCoordinateSystem: 35°52’00’’ N, 01°58’07’’ E; **Identification:** identifiedBy: Ourida Kherbouche-Abrous; **Event:** samplingProtocol: pitfall traps; year: 2019; month: 12; day: 1-15

#### Distribution

**Algerian distribution**: The north part, from the east to the west (Denis 1945b, Bosmans 1985b, Bosmans 2006a, Bouseksou et al. 2015, Touchi et al. 2018, Mansouri et al. 2019).

**Global distribution**: Macaronesia, northern Africa, Europe, Turkey, Caucasus, Russia (Europe to south Siberia), Iran, Kazakhstan, Central Asia. Introduced to Canada, USA, Chile, Argentina, Falkland Is., New Zealand (World Spider Catalog 2024).

### Typhochrestus digitatus

(O. Pickard-Cambridge, 1873)

20D71257-C6F8-5444-BF3E-F2A07D99AE65

#### Materials

- **Type status:** Other material. **Occurrence:** recordedBy: Amina Saadi; individualCount: 1; sex: 1 male; lifeStage: adult; occurrenceID: 1A07654C-1D63-5CD6-BC34-D6307BB5AF26; **Taxon:** scientificNameID: urn:lsid:nmbe.ch:spidersp:013179; scientificName: *Typhochrestusdigitatus* (O. Pickard-Cambridge, 1873); kingdom: Animalia; phylum: Arthropoda; class: Arachnida; order: Araneae; family: Linyphiidae; genus: Typhochrestus; taxonRank: species; taxonomicStatus: accepted; **Location:** higherGeography: North Africa; continent: Africa; country: Algeria; countryCode: DZ; municipality: Theniet El Had; verbatimElevation: 1456 m; verbatimCoordinateSystem: 35°51’18’’ N, 01°59’20’’ E; **Identification:** identifiedBy: Ourida Kherbouche-Abrous; **Event:** samplingProtocol: pitfall traps; year: 2019; month: 10; day: 1-15
- **Type status:** Other material. **Occurrence:** recordedBy: Amina Saadi; individualCount: 1; sex: 1 male; lifeStage: adult; occurrenceID: 8E845FA7-C394-5BF0-BD92-19B577E7D413; **Taxon:** scientificNameID: urn:lsid:nmbe.ch:spidersp:013179; scientificName: *Typhochrestusdigitatus* (O. Pickard-Cambridge, 1873); kingdom: Animalia; phylum: Arthropoda; class: Arachnida; order: Araneae; family: Linyphiidae; genus: Typhochrestus; taxonRank: species; taxonomicStatus: accepted; **Location:** higherGeography: North Africa; continent: Africa; country: Algeria; countryCode: DZ; municipality: Theniet El Had; verbatimElevation: 1470 m; verbatimCoordinateSystem: 35°52’00’’ N, 01°58’07’’ E; **Identification:** identifiedBy: Ourida Kherbouche-Abrous; **Event:** samplingProtocol: pitfall traps; year: 2019; month: 10; day: 1-15

#### Distribution

**Algerian distribution**: North and west of the country (Denis 1937, Bosmans and Abrous 1990).

**Global distribution**: Europe and North Africa (World Spider Catalog 2024).

### Typhochrestus mauretanicus

Bosmans, 1990

68CB3E1E-3A15-5AED-9B2B-BB326FC16BE2

#### Materials

- **Type status:** Other material. **Occurrence:** recordedBy: Badis Bakhouche; individualCount: 5; sex: 5 males; lifeStage: adult; occurrenceID: 0F5D75AE-4713-52BE-AE49-D040802CB2F0; **Taxon:** scientificNameID: urn:lsid:nmbe.ch:spidersp:013188; scientificName: *Typhochrestusmauretanicus* Bosmans, 1990; kingdom: Animalia; phylum: Arthropoda; class: Arachnida; order: Araneae; family: Linyphiidae; genus: Typhochrestus; taxonRank: species; taxonomicStatus: accepted; **Location:** higherGeography: North Africa; continent: Africa; country: Algeria; countryCode: DZ; municipality: Theniet El Had; verbatimElevation: 1456 m; verbatimCoordinateSystem: 35°51’18’’ N, 01°59’20’’ E; **Identification:** identifiedBy: Ourida Kherbouche-Abrous; **Event:** samplingProtocol: hand collecting; year: 2019; month: 4; day: 1-15

#### Distribution

**Algerian distribution**: North-west of the country (Bosmans and Abrous 1990, Bosmans 2008).

**Global distribution**: Morocco, Algeria (World Spider Catalog 2024).

### Walckenaeria erythrina

(Simon, 1884)

9FE4332A-0823-594C-9A3E-685D4D3370BE

#### Materials

- **Type status:** Other material. **Occurrence:** recordedBy: Amina Saadi; individualCount: 1; sex: 1 female; lifeStage: adult; occurrenceID: AFCC91CF-3DCE-5C5E-82B3-C1087449B408; **Taxon:** scientificNameID: urn:lsid:nmbe.ch:spidersp:013287; scientificName: *Walckenaeriaerythrina* (Simon, 1884); kingdom: Animalia; phylum: Arthropoda; class: Arachnida; order: Araneae; family: Linyphiidae; genus: Walckenaeria; taxonRank: species; taxonomicStatus: accepted; **Location:** higherGeography: North Africa; continent: Africa; country: Algeria; countryCode: DZ; municipality: Theniet El Had; verbatimElevation: 1470 m; verbatimCoordinateSystem: 35°52’00’’ N, 01°58’07’’ E; **Identification:** identifiedBy: Ourida Kherbouche-Abrous; **Event:** samplingProtocol: pitfall traps; year: 2019; month: 7; day: 1-15
- **Type status:** Other material. **Occurrence:** recordedBy: Amina Saadi; individualCount: 1; sex: 1 male; lifeStage: adult; occurrenceID: 85521687-EE73-590C-A9ED-6383F766EA1E; **Taxon:** scientificNameID: urn:lsid:nmbe.ch:spidersp:013287; scientificName: *Walckenaeriaerythrina* (Simon, 1884); kingdom: Animalia; phylum: Arthropoda; class: Arachnida; order: Araneae; family: Linyphiidae; genus: Walckenaeria; taxonRank: species; taxonomicStatus: accepted; **Location:** higherGeography: North Africa; continent: Africa; country: Algeria; countryCode: DZ; municipality: Theniet El Had; verbatimElevation: 1470 m; verbatimCoordinateSystem: 35°52’00’’ N, 01°58’07’’ E; **Identification:** identifiedBy: Ourida Kherbouche-Abrous; **Event:** samplingProtocol: pitfall traps; year: 2019; month: 10; day: 1-15

#### Distribution

**Algerian distribution**: North part of the country (Simon 1884, Simon 1926, Bosmans and DeSmet 1993).

**Global distribution**: France (Corsica), Morocco, Algeria, Tunisia (World Spider Catalog 2024).

### Walckenaeria heimbergi

Bosmans, 2007

5B6B92BC-F12B-5B6F-AC46-8BA3CF473D59

#### Materials

- **Type status:** Other material. **Occurrence:** recordedBy: Amina Saadi; individualCount: 2; sex: 2 males; lifeStage: adult; occurrenceID: 6F3A602C-BAC9-5C09-A327-FBE3E6E9550C; **Taxon:** scientificNameID: urn:lsid:nmbe.ch:spidersp:041834; scientificName: *Walckenaeriaheimbergi* Bosmans, 2007; kingdom: Animalia; phylum: Arthropoda; class: Arachnida; order: Araneae; family: Linyphiidae; genus: Walckenaeria; taxonRank: species; taxonomicStatus: accepted; **Location:** higherGeography: North Africa; continent: Africa; country: Algeria; countryCode: DZ; municipality: Theniet El Had; verbatimElevation: 1448 m; verbatimCoordinateSystem: 35°51’30’’ N, 01°59’11’’ E; **Identification:** identifiedBy: Ourida Kherbouche-Abrous; Robert Bosmans; **Event:** samplingProtocol: pitfall traps; year: 2019; month: 2; day: 1-15

#### Distribution

**Algerian distribution**: to date, the species is only known from Théniet El Had National Park.

**Global distribution**: Morocco (World Spider Catalog 2024) and Algeria (First record).

## Discussion

During the sampling period in Théniet El Had National Park (THNP), a total of 167 males and 109 females belonging to 15 genera and 24 species were collected. The genus *Pelecopsis* was the most species-rich. These results are in agreement with the work of Kherbouche-Abrous (2006) in the Djurdjura National Park (Tizi Ouzou, north Algeria) and Mansouri-Tabet (2020) in Chrea National Park (Blida, north Algeria), where they also found that *Pelecospis* is the richest genus.

Amongst the collected species, four (4) are endemic to Algeria and Morocco: *Pelecopsisoranensis*, *P.oujda*, *Typhochrestusmauretanicus* and *Walckenaeriaheimbergi*. Two (2) species are endemic to Algeria: *P.major* and *Sintulapenicilliger*. One (1) to Algeria, Morocco and Tunisia: *Centromerussinuatus* and one (1) to Algeria and Tunisia: *Palliduphanteslabilis*. The remaining species are either Mediterranean, circum-Mediterranean or cosmopolitan.

Comparing our results with previous works in northern Algerian national parks, which are also cedar forests, similarities can be highlighted; Kherbouche-Abrous (2006) collected eight genera and 16 species in the Djurdjura National Park of which nine species were common with our results: *Centromerussinuatus*, *C.succinus*, *Diplocephalusgraecus*, *Mecopisthesmonticola*, *Palliduphanteslabilis*, *P.major*, *Tenuiphantestenuis*, *Typhochrestusdigitatus* and *Walckenaeriaerythrina*. Mansouri-Tabet (2020) sampled eight species belonging to nine genera in the Chrea National Park of which six species were common with those collected in the Théniet El Had National Park (present work): *Agynetapseudorurestris*, *C.succinus*, *Palliduphanteslabilis*, *Pelecopsisinedita*, *Tenuiphantestenuis* and *T.mauretanicus*.

In addition to the species new to the study area, two species *Pelecopsisoujda* Bosmans & Abrous, 1992 and *Walckenaeriaheimbergi* Bosmans 2007, so far known only from Morocco, are new records to Algeria. They were collected only in a pure cedar forest at 1450 m. The first species is only known from a single male collected from Oujda in north-eastern Morocco. This could be the unknown male of *Pelecopsisaureipes* Denis 1962 whose type locality is probably close to Oujda (Bosmans and Abrous 1992). The second species is, until now, only known from the type locality: Douyet, Saiss plain, Fes, Morocco (Bosmans 2007).

Most species were collected in pure or mixed woodlands, open or closed, with no particular preference for any tree species; however, other studies may confirm or refute this assumption. Only *Nematogmussanguinolentus* has been collected in herbaceous vegetation and seems to prefer dry and sunny spots. The majority of the species are stenochronous; they are active during two seasons only, in winter combined with spring or summer.

As this is the first work on Lyniphiidae spiders in Théniet El Had National Park, it is necessary to carry out further field investigations using, in addition to Barber pitfall traps and hand collecting, other sampling methods such as sieving, Japanese umbrella (Ledoux and Canard 1981) consisting of beating or shaking foliage over an upturned umbrella etc., to have a more complete list of the spider community present in this Park. This requires a considerable further sampling effort to obtain more detailed lists of the arthropod fauna in general and the Aranea fauna in particular.

## Supplementary Material

XML Treatment for Agyneta pseudorurestris

XML Treatment for Alioranus pauper

XML Treatment for Centromerus cinctus

XML Treatment for Centromerus sinuatus

XML Treatment for Centromerus succinus

XML Treatment for Diplocephalus graecus

XML Treatment for Gonatium occidentale

XML Treatment for Improphantes decolor

XML Treatment for Lessertia barbara

XML Treatment for Mecopisthes monticola

XML Treatment for Nematogmus sanguinolentus

XML Treatment for Palliduphantes labilis

XML Treatment for Pelecopsis bucephala

XML Treatment for Pelecopsis digitulus

XML Treatment for Pelecopsis inedita

XML Treatment for Pelecopsis major

XML Treatment for Pelecopsis oranensis

XML Treatment for Pelecopsis oujda

XML Treatment for Sintula penicilliger

XML Treatment for Tenuiphantes tenuis

XML Treatment for Typhochrestus digitatus

XML Treatment for Typhochrestus mauretanicus

XML Treatment for Walckenaeria erythrina

XML Treatment for Walckenaeria heimbergi
